# Ameliorative effects of mulberry fruit anthocyanin extract on gut microbiota and liver metabolites in high-fat and high-cholesterol diet-fed ApoE−/− mice

**DOI:** 10.3389/fnut.2026.1780996

**Published:** 2026-03-18

**Authors:** Tala Shi, Xinyuan Li, Shuo Wen, Wei Wang, Shuai Hou, Wenqing Jiang, Wei Mi, Zhiyong Hu, Wu Lian, Sha Liu, Peng Lu

**Affiliations:** School of Public Health, Binzhou Medical University, Yantai, China

**Keywords:** cyanidin-3-O-glucoside, gut microbial, high fat and high cholesterol diet, liver metabolites, mulberry fruit anthocyanin extract

## Abstract

**Aim:**

This study aims to investigate the effects of mulberry anthocyanin (MA) in high-fat and high-cholesterol (HFHC) diet-fed ApoE−/− mice.

**Methods:**

ApoE−/− mice were randomly divided into control (ACON), mulberry fruit anthocyanin extract (MFAE), cyanidin-3-glucoside (C3G) group 1 (C3GT), and C3G group 2 (C3GP). After 7 weeks of HFHC diet feeding and following 2–3 weeks of treatment, samples were collected and analyzed.

**Results:**

The C3GT group significantly decreased low-density lipoprotein (7.3 ± 1.5 mmol/L) and interleukin-1β (355.4 ± 41.7 pg./mL) levels. Moreover, the MFAE (636.3 ± 90.7 pg./mL), C3GT (611.5 ± 65.4 pg./mL), and C3GP (757.5 ± 47.6 pg./mL) significantly increased glutathione peroxidase (GSH-PX) levels compared with those in the ACON group. The MA treatments significantly increased the number of *Anaerotruncus*, *Tyzzerella*, and *Butyricicoccus,* while decreasing the abundance of *Sphingomonas*, *Odoribacter*, and *Rikenella.* The MA intervention not only decreased the adenosine-5′-triphosphate (ATP) and indole-3-butyric acid but also upregulated the Pg36:3 and glutamine.

**Conclusion:**

MA treatment may attenuate AS-associated risk factors by decreasing inflammatory factor-related gut microbial genera. The mechanism may be related to regulating liver glutamine, ATP, and related metabolic pathways.

## Introduction

Atherosclerosis (AS) is an inflammatory disease and pathological condition that induces cardiovascular disease (CVD). Smoking, lack of physical activity, and an unhealthy diet are risk factors for AS, which account for approximately 80% of CVD cases (1). A high-fat diet (HFD) elevates low-density lipoprotein (LDL) levels, causing lipid accumulation, oxidative stress damage, chronic inflammation, foam-cell formation, and death (2). Consuming plant-based foods, including vegetables and fruits, is inversely associated with CVD risk (3). Several epidemiological and cohort studies have revealed that the regular consumption of flavonoid-rich foods and beverages decreases blood triglycerides (TG), total cholesterol (TC), and LDL cholesterol (LDL-C) levels, as well as the risk of CVD mortality (4, 5).

Anthocyanins, glycosylated polyhydroxy and polyethoxy derivatives of flavonoids, exhibit vasoprotective properties (6). Notably, *in vitro* studies have demonstrated that anthocyanins and their metabolites exert antioxidant, anti-inflammatory, antiatherogenic, and vasodilating effects (7), and their intake can improve functional vascular health *in vivo* (8). Notably, anthocyanin consumption increases high-density lipoprotein cholesterol concentrations and decreases LDL-C concentrations in a double-blind, randomized, placebo-controlled human trial (9). Moreover, the bioactivity of anthocyanins has been associated with gut microbiota (10). The gut microbiome can metabolize anthocyanins, thereby providing smaller, more bioavailable end products that positively affect the physiological processes of the host, function as signaling molecules, and serve as substrates for numerous metabolic reactions (11).

Bacterial DNA in AS plaques indicates an association between gut microbiota and AS (12, 13). Studies have reported that *Bifidobacteria, Lactobacillus* (14), *Roseburia intestinalis,* and *Akkermansia muciniphila* (15) exert protective effects against AS by reducing inflammatory markers and AS plaques. The genus *Roseburia* has also been shown to be promising for AS lesion treatment by producing butyrate, which is inversely associated with coronary artery calcium (CAC) progression, an independent risk factor for AS (16). In addition, a recent study reported that microbial metabolites of tryptophan, including indole-3-propionic acid, indole-3-acetic acid, and indole-3-carboxaldehyde, were significantly decreased in the arteriosclerosis model. However, gut microbiota also metabolized anthocyanins into syringic acid, vanillic acid, phloroglucinol aldehyde, phloroglucinol acid, and gallic acid, which can exhibit health-enhancing effects associated with anthocyanins (3). For example, gallic acid can be an effective protector against monocyte recruitment in inflammatory vessels and may be instrumental in preventing AS lesion development. Protocatechuic acid reduced plasma TNF-*α*, nitric oxide, and hepatic malondialdehyde levels (17). However, studies have shown that the ratio of Firmicutes to Bacteroidetes in patients with AS is notably higher than that in controls, and the genus *Collinsella* has been observed in AS plaques. Notably, the occurrence of the genera in *Chryseomonas* and *Helicobacter* is higher in AS patients compared with healthy adults. Moreover, the genera *Sporogenes*, *Collinsella,* and *Actinobacteria* have been shown to promote AS progression (14, 15, 18). Furthermore, studies revealed that gut microbial metabolites, including trimethylamine, can be converted to trimethylamine-N-oxide (TMAO) by the liver, which is involved in the relationship between intestinal microbiota and adverse cardiovascular events in humans and increase the risk of AS (19).

The anthocyanin extract of mulberry fruit exhibits multiple bioactive functions, including antioxidant (20), neuroprotective (21), antiatherogenic (22), immunoregulatory (23), antitumor, antihyperglycemic, and antihyperlipidemic activities (24); however, its therapeutic effects and mechanisms remain unclear. Mulberry anthocyanin (MA) primarily comprises cyanidin-3-glucoside (C3G), cyanidin-3-rutinoside (C3R), and pelargonidin-3-glucoside (P3G). Notably, C3G can improve insulin resistance in adipose tissue, and the liver exerts a protective role against H_2_O_2_ and TNF-*α*-induced insulin resistance (25, 26). Furthermore, C3G prevents hyperglycemia-induced hepatic oxidative damage by activating glutathione synthesis and attenuating oxidative stress in mice (27). However, investigations at the level of lipid metabolism are insufficient to illustrate its effects on AS.

The present study aims to assess the effects of MFAE, a semi-purified anthocyanin extracted from dried mulberry fruits, and its primary component, C3G, on the gut microbial structure and liver metabolism in a high-fat and high-cholesterol diet (HFHC)-induced ApoE−/− mouse model.

## Materials and methods

### Preparation of anthocyanin extract from mulberry fruit

To prepare the mulberry fruit anthocyanin extract (MFAE), freeze-dried mulberry fruits were crushed into a powder using a grinder, sieved through a 0.216-mm filter, placed in a sealed dark bottle, and stored at 4 °C. Subsequently, 50 g of mulberry powder was mixed with 1,000 mL of an acidified (pH 3.0, 0.1% hydrochloric acid‌) 70% ethanol solution and sonicated at 40 °C and 200 W for 40 min. The solution was then centrifuged at 1500*× g* for 15 min, and the ethanol in the supernatant was removed via evaporation at 37 °C, 150 *× g*. Macroporous resin was used to purify the solution at a ratio of 1 g:50 mL (resin-to-supernatant). A filter was used to soak the adsorbed macroporous resin from the 70% ethanol solution. Next, the solution was placed in a shaking incubator for 12 h until the color of the solution remained constant, and the macroporous resin was filtered out. Following rotary evaporation, the solution was divided into centrifuge tubes, freeze-dried to a powder state, and stored at –20 °C.

The MFAE anthocyanin monomers were determined by a Thermo Q Exactive high-performance liquid chromatography-mass spectrometry (HPLC–MS) system. Methanol-containing acetonitrile (A) and a 0.1% formic acid aqueous solution (B) were used as the HPLC mobile phase. MS analysis conditions were set as follows: run time, 10 min; full MS resolution, 70,000; AGC target, 3e6; maximum IT, 100 ms; scan range, 100–2000 m/z; dd-MS/dd-SIM resolution, 17,500; AGC target, 1e5; maximum IT, 50 ms; isolation window, 4.0 m/z; and collision energy, 20/30/40 in NCE mode.

### Experimental design and animal care

**Figure 1:**
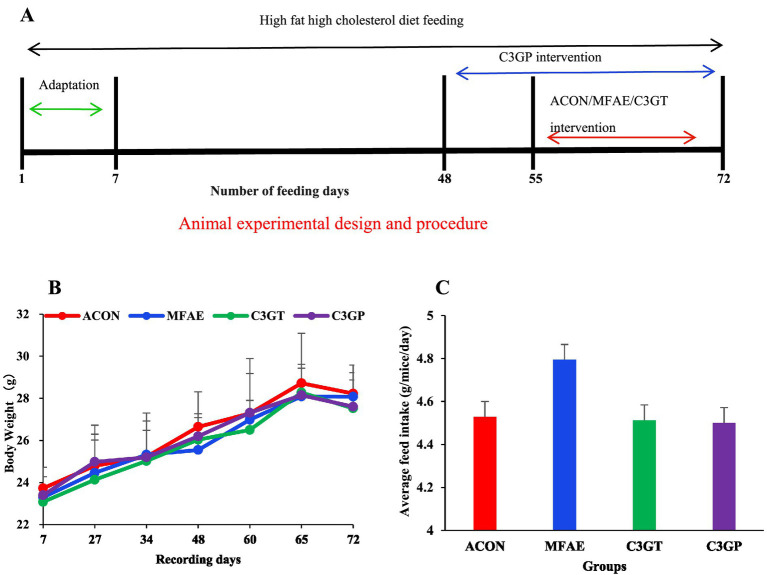
**(A)** Animal experimental design and procedure; **(B)** MA effect on body weight and **(C)** feed intake during different experimental periods (*n =* 6). ACON, ApoE^−/−^ control group (red line/bar); MFAE, mulberry fruit anthocyanin extract treatment group (blue line/bar); C3GT, 2-week cyanidin-3-glucoside intervention group (green line/bar); C3GP, 3-week cyanidin-3-glucoside intervention group (purple line/bar).

In total, 32 (8-week-old) male ApoE**−**/**−** mice (Hangzhou Ziyuan Experimental Animal Technology Co., Ltd., Zhejiang, SCXK (zhe) 2019–0004) were housed in a pathogen-free facility (8 mice/cage, 25 ± 3 °C, 55 ± 5% relative humidity, and a 12/12-h light/dark cycle) and were provided free access to tap water and the HFHC diet (the feed, which contained 21% lard and 0.15% cholesterol, was purchased from Beijing Keao Xieli Feed Co., Ltd., Beijing, China). After 1 week of adaptation, mice were randomly divided into four groups: control, MFAE, C3G group 1 (C3GT), and C3G group 2 (C3GP). To compare the different intervention period effects of the C3G, mice in the C3GP group were intragastrically administered 25 mg/kg·d of C3G (Caoyuankang Biotechnology Co., Ltd., Chengdu, China) after feeding a HFHC diet for 7 weeks. However, the mice in the ACON, MFAE, and C3GT groups were intragastrically administered 20 mL/kg·d saline solution, 200 mg/kg·d MFAE, and 25 mg/kg·d C3G after feeding the HFHC diet for 8 weeks, respectively. The experimental procedure is shown in Figure 1A. Following a 2- or 3-week intervention, all mice were anesthetized by inhalation of 3–5% ether (2–3 cotton balls immersed in analytical grade ether, placed them at the bottom of a transparent sealed anesthesia chamber, and put the mouse into the chamber, tighten the lid, wait for 1–2 min); when the mouse corneal reflex disappeared and it was unable to turn over by itself, the mice were removed and blood samples immediately collected from the orbital veins, followed by cervical dislocation for euthanasia. After 2 min without a blink reflex, death was confirmed by lightly pressing the mouse’s chest to verify the absence of a heartbeat. The stool samples were collected in a sterile tube under clean bench conditions by cutting the colon and stored at −80 °C prior to analysis. The liver tissues were washed in phosphate-buffered saline and fixed in formalin for histological examination. All experimental protocols complied with the care and use of laboratory animals in scientific investigations and were approved by the Ethics of Animal Use Research Committee of Binzhou Medical University (2023–372).

### Biochemical assay of blood samples

The serum samples were collected after centrifugation at 3500 rpm for 15 min, 4 °C. The levels of TC, LDL-C, interleukin-1β (IL-1β), glutathione peroxidase (GSH-PX), and tumor necrosis factor-*α* (TNF-α) were assessed using an enzyme-linked immunosorbent assay (ELISA) kit (Shanghai Jianglai Biological Technology Co., Ltd.) following the manufacturer’s instructions.

### Histological examination of the liver tissue

The fresh tissues were fixed with 4% paraformaldehyde, dehydrated using an ethanol gradient, cleared in xylene, and embedded in paraffin. Subsequently, the tissue blocks were cut into 5 μm sections, stained with hematoxylin and eosin (H&E), and observed using light microscopy (Nikon Eclipse E100, Japan).

### Gut microbiota analysis

The fecal samples’ whole-genome DNA was extracted, and DNA purity was determined using 1% agarose gel electrophoresis. DNA samples were diluted to 1 ng/μL and amplified using polymerase chain reaction (PCR) targeting the V3 + V4 region. The PCR products were detected using 2% agarose gel electrophoresis and purified using a Qiagen Gel Recovery Kit, following the instructions. Finally, the sequencing library was constructed using a TruSeq^®^ DNA PCR-Free sample preparation kit (Illumina, USA). Following Qubit quantification and detection, the library was sequenced using a NovaSeq 6,000 PE250. The Chao1 and Faith’s PD indices were used to evaluate bacterial richness and diversity. Linear discriminant analysis (LDA) was performed to identify the key bacterial taxa that differed among the groups (log_10_ > 2.0). The microbial functional prediction was performed by the Phylogenetic Investigation of Communities by Reconstruction of Unobserved States (PICRUSt) analysis.

### Liver metabolic profile analysis

The metabolites of the liver tissues were extracted with a methanol/acetonitrile (1:1, v/v) solution, and the vacuum-dried supernatant was re-dissolved and applied for Liquid Chromatography-Mass Spectrometry (LC–MS) analysis. For hydrophilic interaction chromatography separation, samples were analyzed using a 2.1 mm × 100 mm ACQUITY UPLC BEH Amide 1.7 μm column (Waters, Ireland). In both ESI positive and negative modes, the mobile phase contained 25 mM ammonium acetate and 25 mM ammonium hydroxide in water (A) and acetonitrile (B). The gradient was 95% B for 0.5 min and was linearly reduced to 65% in 6.5 min, and then was reduced to 40% in 1 min and kept for 1 min, and then increased to 95% in 0.1 min, with a 3 min re-equilibration. In the extracted ion features, only the variables with more than 50% of the nonzero measurement values in at least one group were kept. Compound identification of metabolites was performed by comparing the accuracy of the m/z value (<10 ppm) and MS/MS spectra with an in-house database established with available authentic standards.

### Statistical analysis

Body weights and biochemical data were analyzed using SPSS 24.0 software, and data were presented as the mean ± standard deviation (SD). One-way analysis of variance (ANOVA) with Tukey’s honest significant difference comparison was used. Differences between the gut microbiota and liver metabolites were assessed using one-way ANOVA or the Kruskal–Wallis (KW) rank-sum test, and Dunn’s test (R package) was conducted to assess differences between the groups. Spearman’s correlation analysis was conducted using the R package (v4.0.0) to evaluate the relationship between bacterial genera and physiological parameters. Statistical significance was set at *p* < 0.05.

## Results

### MFAE and C3G content and composition

The content of semi-purified anthocyanins in the experimental mulberry fruit was 650 mg/100 g. The LC–MS results were compared with previously published fragment information, and 17 types of anthocyanins were identified. The MS/MS fragmentation ions, mass-to-charge ratio (*m/z*), and other component information are provided in the Supplementary Table S1.

### Effects on body weight and food intake

As shown in Figures 1B,C, the body weight and feed intake of the mice were recorded. The HFHC diet increased body weight, but there was no significant difference in body weight and feed consumption among groups.

### Effects on blood biochemical indices

Compared with the ACON group, the MFAE, C3GT, and C3GP groups exhibited decreased LDL-C and IL-1β levels and significantly increased GSH-PX levels (*p* < 0.05). However, TC and TNF-*α* were not significantly changed following the different MA interventions (Table 1).

**Table 1:** Changes in serum biochemical indices.

Groups	TC (mmol/L)	LDL-C (mmol/L)	IL-1β (pg/mL)	TNF-α (pg/mL)	GSH-PX (pg/mL)
ACON	13.8 ± 2.1	14.4 ± 3.4	418.5 ± 31.3	961.3 ± 107.2	429.4 ± 18.2
MFAE	13.4 ± 3.0	10.8 ± 2.1	416.3 ± 57.7	899.2 ± 71.2	636.3 ± 90.7^*^
C3GT	17.8 ± 2.5	7.3 ± 1.5^*#^	355.4 ± 41.7^*^	1060.0 ± 77.8^#^	611.5 ± 65.4^*^
C3GP	17.9 ± 6.1	9.7 ± 4.4	356.6 ± 41.4^*^	985.3 ± 95.8	757.5 ± 47.6^*&^
*F*	2.053	4.375	7.081	4.140	23.777
*df*	18	16	19	18	16
*P*	0.150	0.025	0.003	0.025	0.000

*P < 0.05, compared with the ACON group; ^#^*p* < 0.05, compared with the MFAE group; ^&^*p* < 0.05, compared with the C3GT group (one-way ANOVA–Tukey HSD).

### Effects on liver tissue pathology

**Figure 2:**
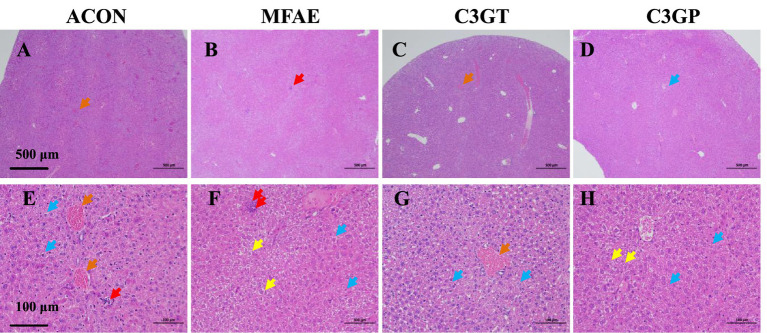
Effects of MA treatment on liver histopathological alterations examined by H&E staining (*n =* 3, **A–D**, magnification: 40×; ruler, 500 μm; **E–H**, magnification: 200×; ruler, 100 μm;). In **(A–H)**, orange arrows indicate congestion in liver blood vessels; red arrows, lymphocyte infiltration; blue arrows, watery degeneration of liver cells; yellow arrows, ballooning of liver cells. ACON, ApoE^−/−^ mice control group; MFAE, mulberry fruit anthocyanin extract treatment group; C3GT, 2-week cyanidin-3-glucoside intervention group; C3GP, 3-week cyanidin-3-glucoside intervention group.

Pathological examination of the liver revealed that the ACON group exhibited mild watery hepatocyte degeneration, inflammatory cell infiltration, and minimal congestion. Compared with the ACON group, an increased range of watery hepatocyte degeneration, along with the disappearance of congestion and inflammation, was observed in the MFAE, C3GT, and C3GP groups. Notably, a few samples from the C3GT and MFAE groups did not exhibit inflammatory responses or watery degeneration (Figures 2A–H).

### Effects on gut microbial diversity

**Figure 3:**
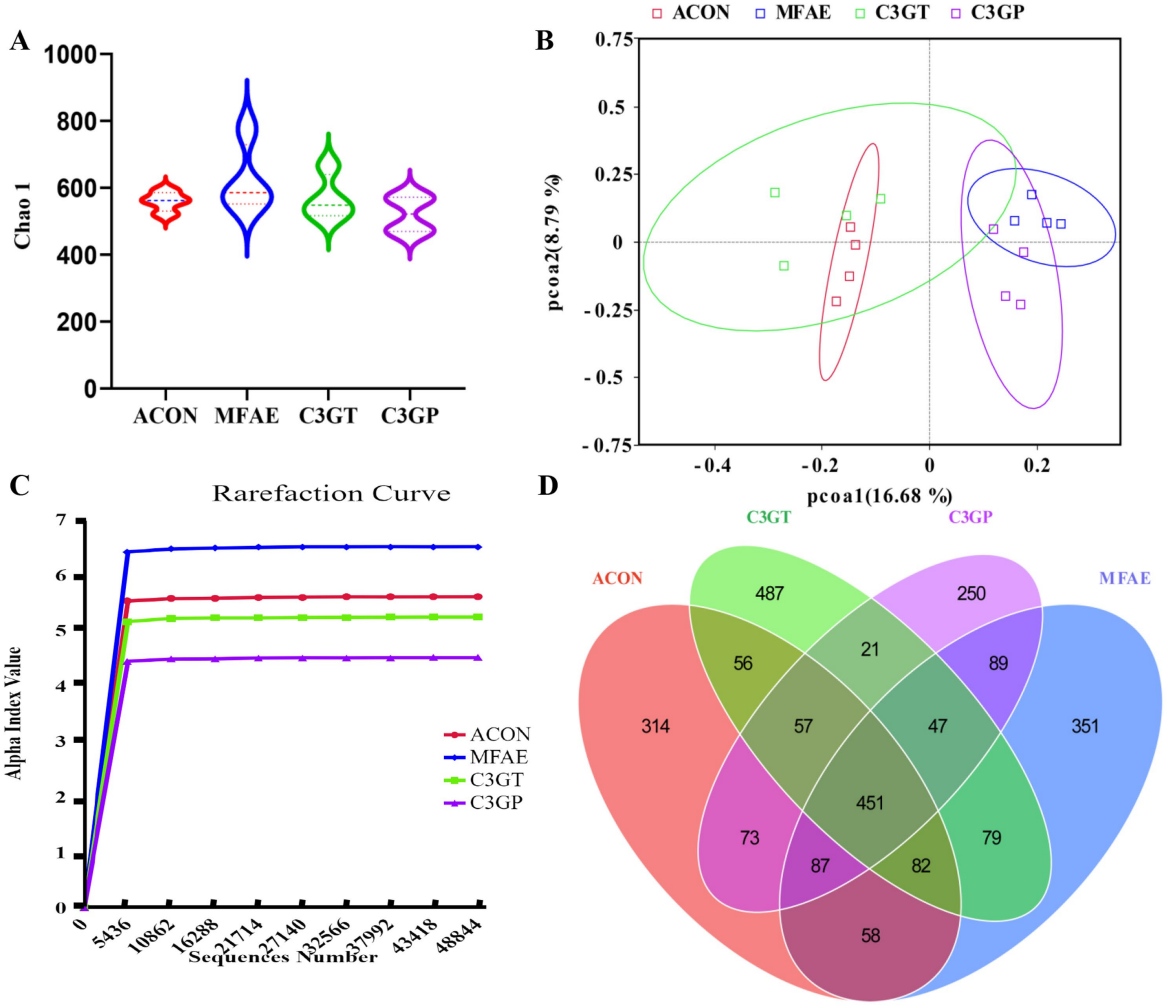
Effects of MA treatment on microbial diversity analysis. **(A)** Chao1 *α*-diversity analysis; **(B)** PCoA *β*-diversity analysis; **(C)** Rarefaction curve; **(D)** Venn diagram (*n =* 4). ACON, ApoE^−/−^ mice control group; MFAE, mulberry fruit anthocyanin extract treatment group; C3GT, 2-week cyanidin-3-glucoside intervention group; C3GP, 3-week cyanidin-3-glucoside intervention group.

Following quality filtration, the samples generated an average of 117,722 ± 7,065 reads, and valid sequences were clustered as 2,609 amplicon sequence variants (ASVs). Alpha diversity analysis, including the Chao1 and Shannon indices, revealed no significant differences between groups. However, the principal coordinate beta diversity analysis revealed distinct features between the groups. The rarefaction curve and Venn diagram displayed the highest specific number of ASVs in the C3GT group, followed by the ACON, C3GP, and MFAE groups. These results indicated that MA intervention did not affect inter-group richness but changed the gut microbial diversity among the groups (Figures 3A–D).

### Effects on the taxonomic composition of gut microbiota

**Figure 4:**
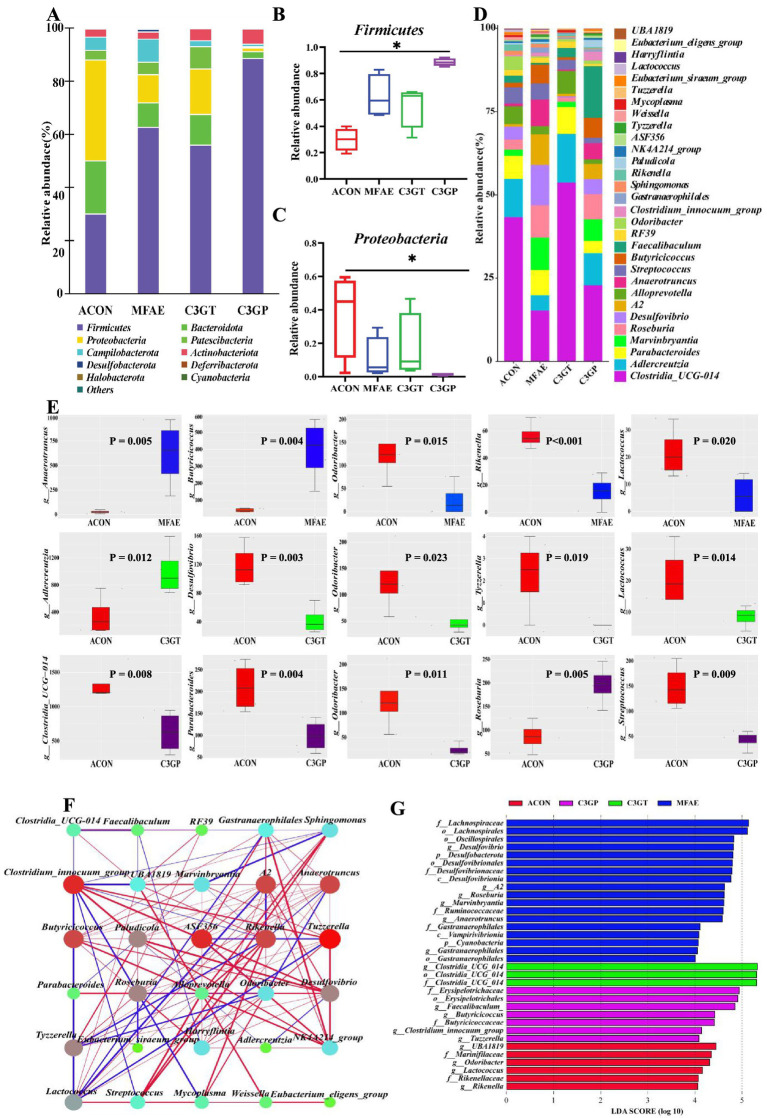
Effects of MA treatment on the taxonomic composition of gut microbiota. **(A–C)** Phylum-level diversity; **(D)** Significantly different bacterial genera between groups (one-way ANOVA and R package Dunn’s test analysis); **(E)** Top five significant different bacterial genera between groups (metastat analysis between groups); **(F)** Spearman correlation analysis for significantly different genera (red line: positive correlation, blue line: negative correlation, line width corresponds to correlation; circle size corresponds to relative abundance degree, different circle colors indicate different phyla); **(G)** LEfSe (LDA > 4) analysis (*n =* = 4). ACON, ApoE^−/−^ mice control group; MFAE, mulberry fruit anthocyanin extract treatment group; C3GT, 2-week cyanidin-3-glucoside intervention group; C3GP, 3-week cyanidin-3-glucoside intervention group.

At the phylum level, 20 bacterial phyla were annotated into four different groups. Compared with the ACON group, the relative abundances of Firmicutes increased by 52.2, 46.4, and 66.2%, but the relative abundance of Proteobacteria decreased by 72.0, 54.9, and 96.3% in the MFAE, C3GT, and C3GP groups, respectively (Figures 4A–C).

At the genus level, 223 genera were annotated, of which 30 bacterial genera were significantly different between the groups. Compared with the ACON group, MFAE treatment increased the abundances of the six genera, including *Anaerotruncus* (95%), *Tyzzerella* (90%), *Butyricicoccus* (89%), *Harryflintia* (70%), *NK4A214_group* (70%), and *Paludicola* (50%), but decreased *Eubacterium_siraeum* (87%), *Odoribacter* (79%), *Rikenella* (72%), and *Lactococcus* (71%). C3GT treatment increased *Adlercreutzia* (90%) and *Eubacterium_eligens* (65%) and decreased *Desulfovibrio* (63%) and *Lactococcus* (63%). C3GP treatment increased 10 bacterial genera, including *Tyzzerella* (86%), *Faecalibaculum* (83%), *A2* (80%), *Anaerotruncus* (80%), *Clostridium_innocuum* (78%), *Tyzzerella* (73%), *Butyricicoccus* (86%), *Marvinbryantia* (65%), *ASF356* (63%), and *Roseburia* (55%); and decreased 10 bacterial genera, including *Lactococcus* (93%), *Sphingomonas* (81%), *Odoribacter* (80%), *Alloprevotella* (78%), *Rikenella* (75%), *Mycoplasma* (73%), *Streptococcus* (71%), *RF39* (63%), *Parabacteroides* (54%), and *Clostridia_UCG-014* (53%) (Figure 4D). The metastatic analysis revealed the top five significantly different bacterial genera between the two groups, the abundance of *Odoribacter* being significantly decreased by MFAE, C3GT, and C3GP treatments (Figure 4E). In addition, Spearman’s correlation analysis of significantly different bacterial genera revealed that the increased bacterial genera *Anaerotruncus*, *Tyzzerella*, and *Butyricicoccus* were negatively correlated with *Lactococcus*, *Odoribacter*, and *Rikenella*, which were the decreased bacterial genera (Figure 4F).

Furthermore, the LDA effect size (LDA > 4) results revealed *UBA-1819*, *Odoribacter*, *Lactococcus*, and *Rikenella* as the characteristic bacterial genera in the ACON group. Conversely, *Desulfovibrio, Roseburia*, and *Marvinbryantia* were the dominant bacterial genera in the MFAE group, whereas *Clostridia_UCG-014, Faecalibaculum*, and *Butyricicoccus* were the dominant bacterial genera in the C3GT and C3GP groups, respectively. These results indicated that different types of MA interventions can increase beneficial bacterial genera and reduce the relative abundance of pathogenic or opportunistic bacteria (Figures 4F,G).

### Effects on microbial functions

**Figure 5:**
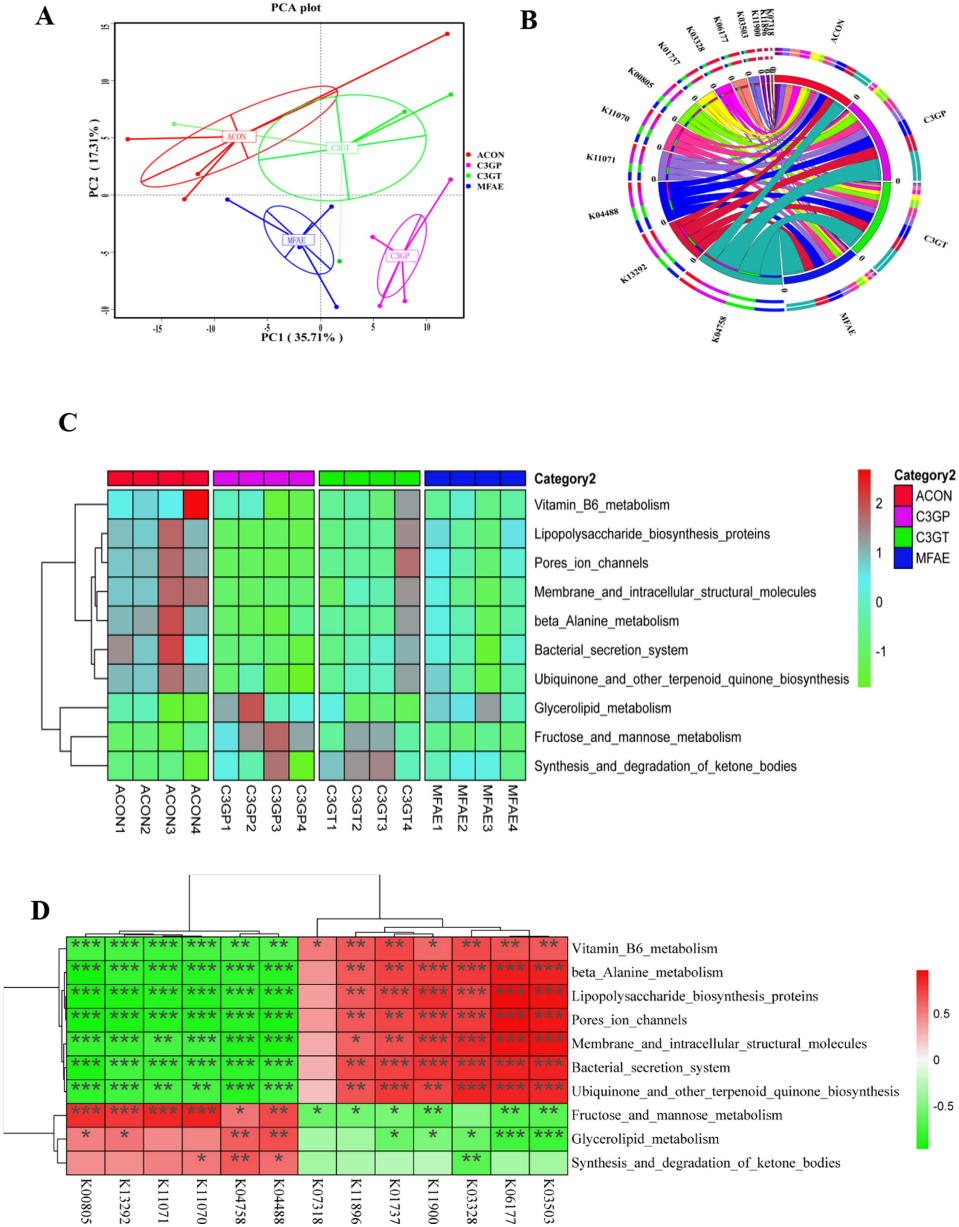
PICRUSt functional prediction analysis of gut microbiota with the KEGG database. **(A)** PCA analysis; **(B)** 13 significantly affected genes by MA treatment (one-way ANOVA and R package Dunn’s test analysis) (*n =* 4); **(C)** Significantly different and shared pathways within groups [Kruskal–Wallis (KW) rank-sum test]; **(D)** Correlation analysis between different pathways and genes within groups. ACON, ApoE^−/−^ mice control group; MFAE, mulberry fruit anthocyanin extract treatment group; C3GT, 2-week cyanidin-3-glucoside intervention group; C3GP, 3-week cyanidin-3-glucoside intervention group.

The functional prediction analysis using PICRUSt of the gut microbiota was aligned with the Kyoto Encyclopedia of Genes and Genomes (KEGG) databases. At the KEGG level, 3,283 pathways were annotated, and differences between the groups were clustered using principal component analysis (PCA) (Figure 5A). Compared with the ACON group, MFAE treatment upregulated 11 pathways, including sporulation (51.8%), glycerollipid metabolism (14%), fatty acid biosynthesis (9.9%), the pentose phosphate pathway (10.0%), and porphyrin and chlorophyll metabolism (26.3%), whereas it downregulated 15 pathways, including membrane and intracellular structural molecules (40.4%), lipopolysaccharide (LPS) biosynthesis proteins (35.3%), ubiquinone and other terpenoid–quinone biosynthesis (35.0%), pore ion channels (33.6%), and *β*-alanine metabolism (18.5%). C3GT treatment upregulated fructose and mannose metabolism (22.6%), synthesis and degradation of ketone bodies (42.5%), pathogenic *Escherichia coli* infection (87.0%), and the spliceosome (47.3%). C3GP treatment upregulated 14 pathways, of which the top five were linoleic acid metabolism (52%), bisphenol degradation (47%), polycyclic aromatic hydrocarbon degradation (47%), fructose and mannose metabolism (33%), and chloroalkane and chloroalkene degradation (32%). Conversely, C3GP treatment downregulated 37 pathways, including pertussis (93%), glycan biosynthesis and metabolism (88%), LPS biosynthesis proteins (71%), LPS biosynthesis (78%), and steroid biosynthesis (70%) (Supplementary Figure S1). MFAE and C3G treatments upregulated glycerolipid metabolism, the synthesis and degradation of ketone bodies, and fructose and mannose metabolism, whereas they decreased the bacterial secretion system, membrane, and intracellular structural molecules, LPS biosynthesis proteins, pore ion channels, *β*-alanine metabolism, ubiquinone and other terpenoid–quinone biosynthesis, and vitamin B6 metabolism pathways (Figure 5C).

At KEGG level 4, MFAE and C3G treatments affected 13 genes, upregulating six genes, including ferrous iron transport protein A (K04758), phosphatidylglycerol: prolipoprotein diacylglycerol transferase (K13292), nitrogen fixation protein (NifU) and related proteins (K04488), spermidine/putrescine transport system permease protein (K11071/K11070), and trans-hexaprenyl transtransferase hepatorenal diphosphate synthase (K00805). Conversely, these treatments downregulated seven genes, including 6-pyruvate tetrahydrobiopterin synthase (K01737; EC:4.2.3.12), polysaccharide transporter, PST family (K03328), tRNA pseudouridine32 synthase/[EC:5.4.99.28] (K06177), DNA polymerase V [EC:3.4.21.-] (K03503), type VI secretion system protein, ImpC (K11900), type VI secretion system protein, ImpG (K11896), and adenine-specific DNA methyltransferase (K07318) (Figure 5B).

Further correlation analysis between predicted genes and pathway analysis revealed that upregulated genes were positively correlated with increased pathways, while downregulated genes had a negative correlation (Figure 5D).

### Effects on liver metabolites

The non-targeted metabolic analysis of the liver tissues identified 1820 metabolites in the four experimental groups, with 997 and 823 in the positive and negative ion modes, respectively. The top five metabolites with a higher proportion at the superclass level were lipids and lipid-like molecules (22.582%), organic acids and their derivatives (22.033%), benzenoids (12.473%), organoheterocyclic compounds (12.473%), and a group of undefined metabolites (9.341%). The top five class-level metabolites were carboxylic acids and their derivatives (18.35%), a group of undefined metabolites (9.34%), benzene and its substituted derivatives (9.011%), organic oxygen compounds (8.022%), and fatty acyls (7.033%) (Supplementary Figures S2A,B).

**Figure 6:**
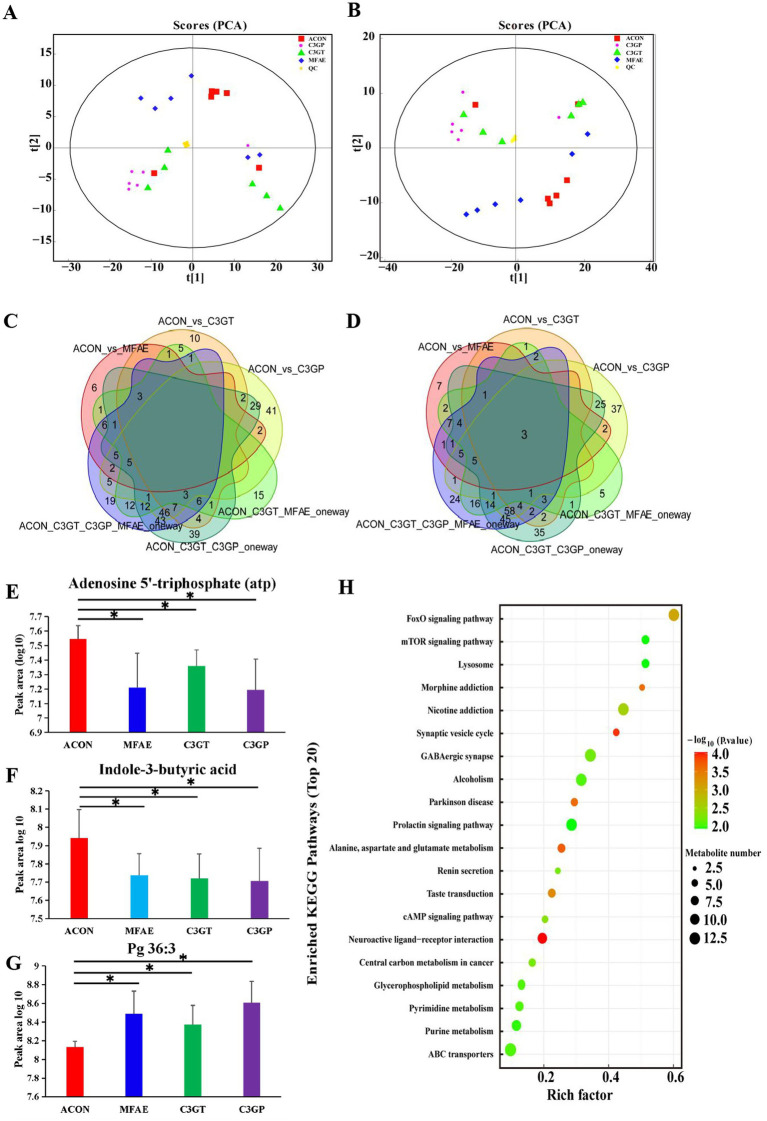
MA effects on liver metabolites. **(A,B)** PCA analysis of the metabolites; **(C,D)** group differential metabolite numbers on positive and negative ion modes; **(E–G)** shared significantly different metabolites within ACON, MFAE, C3GT, and C3GP (*, *p* < 0.05, R package Dunn’s test analysis); **(H)** op 20 significantly different metabolic pathways between ACON and C3GP (R package Dunn’s test analysis) (*n =* 6). ACON, ApoE−/− mice control group; MFAE, mulberry fruit anthocyanin extract treatment group; C3GT, 2-week cyanidin-3-glucoside intervention group; C3GP, 3-week cyanidin-3-glucoside intervention group.

The PCA of the metabolites in ESI + mode is shown in Figures 6A,B. Except for C3GT, the separation between the groups and QC samples was closely distributed and highly correlated, indicating that the entire detection process was stable (Supplementary Figure S3). Furthermore, the orthogonal partial least squares-discriminant analysis (OPLS-DA) results confirmed a significant separation between the two groups in the positive and negative ion modes (Supplementary Figure S4). To prevent overfitting of the OPLS-DA model, its quality was examined using the 200-response reciprocity test method, and the results indicated that the model was reliable and accurate in the positive and negative ion modes (Supplementary Figure S5).

The differential metabolite analysis revealed that, compared with the ACON group, the MFAE group exhibited 32 and 38 significantly different metabolites in the positive and negative ion modes, respectively. Moreover, the C3GT group had 43 and 19 different metabolites in the positive and negative ion modes, respectively. Furthermore, the C3GP group had 148 and 142 different metabolites in the positive and negative ion modes, respectively (Figures 6C,D and Supplementary Figure S6). Among them, adenosine-5′-triphosphate (ATP; nucleosides, nucleotides, and analogs) and indole-3-butyric acid (organoheterocyclic compounds and indoles) were significantly downregulated, whereas Pg36:3 (lipids and lipid-like molecules and glycerophosphoglycerols) was significantly upregulated in the MFAE, C3GT, and C3GP groups compared with the ACON group (Figures 6E–G). Spearman correlation analysis between the different metabolites revealed a positive correlation between ATP and indole-3-butyric acid (Supplementary Figure S7).

KEGG was used to evaluate the enrichment analysis of the pathways and compare the signal transduction pathways involved in the significantly differentiated metabolites between the groups. Notably, 35 pathways (impact value ˃ 0.05) were selected for further illustration by performing the Dunn test to assess differences between the groups. Compared with the ACON group, the MFAE group exhibited four different metabolic pathways, including vitamin B6 metabolism, phosphatidylinositol signaling system, hypertrophic cardiomyopathy (HCM), and lysosome pathway. In these four pathways, the metabolites ATP, D-myo-inositol-1,3-diphosphate, and myo-inositol were significantly affected. The C3GT group exhibited 12 different pathways, including the lysosome pathway, *β*-alanine metabolism, HCM, prolactin signaling pathway, synaptic vesicle cycle, butanoate metabolism, retrograde endocannabinoid signaling, and neuroactive light receptor interaction. In these pathways, only five metabolites were significantly affected, including 3-hydroxybutyric acid, gamma-aminobutyric acid, diacetyl, 1-stearoyl-2-oleoyl-sn-glycerol 3-phosphocholine, and ATP. The C3GP group exhibited 31 different pathways, of which the top five were neuroactive ligand–receptor interaction, synaptic vesicle cycle, alanine, aspartate, and glucose metabolism; morphine addiction; and Parkinson’s disease (Supplementary Figure S8). In this pathway, 30 metabolites were significantly affected, including glutamic acid, adenosine-5′-monophosphate (AMP), glutamine, gamma-aminobutyric acid, acetylcholine, adenosine-5′-diphosphate (ADP), and 3-phospho-D-glycerate, which are related to 41, 20, 17, 16, 13, 12, and 11 types of metabolic pathways, respectively. Moreover, the MFAE, C3GT, and C3GP groups significantly affected the lysosome pathway. In this pathway, ATP was the significantly affected metabolite and was related to 20 other significantly affected metabolic pathways (Figure 6H).

### Correlation analyses of bacterial genera, biochemical indicators, and metabolites

**Figure 7:**
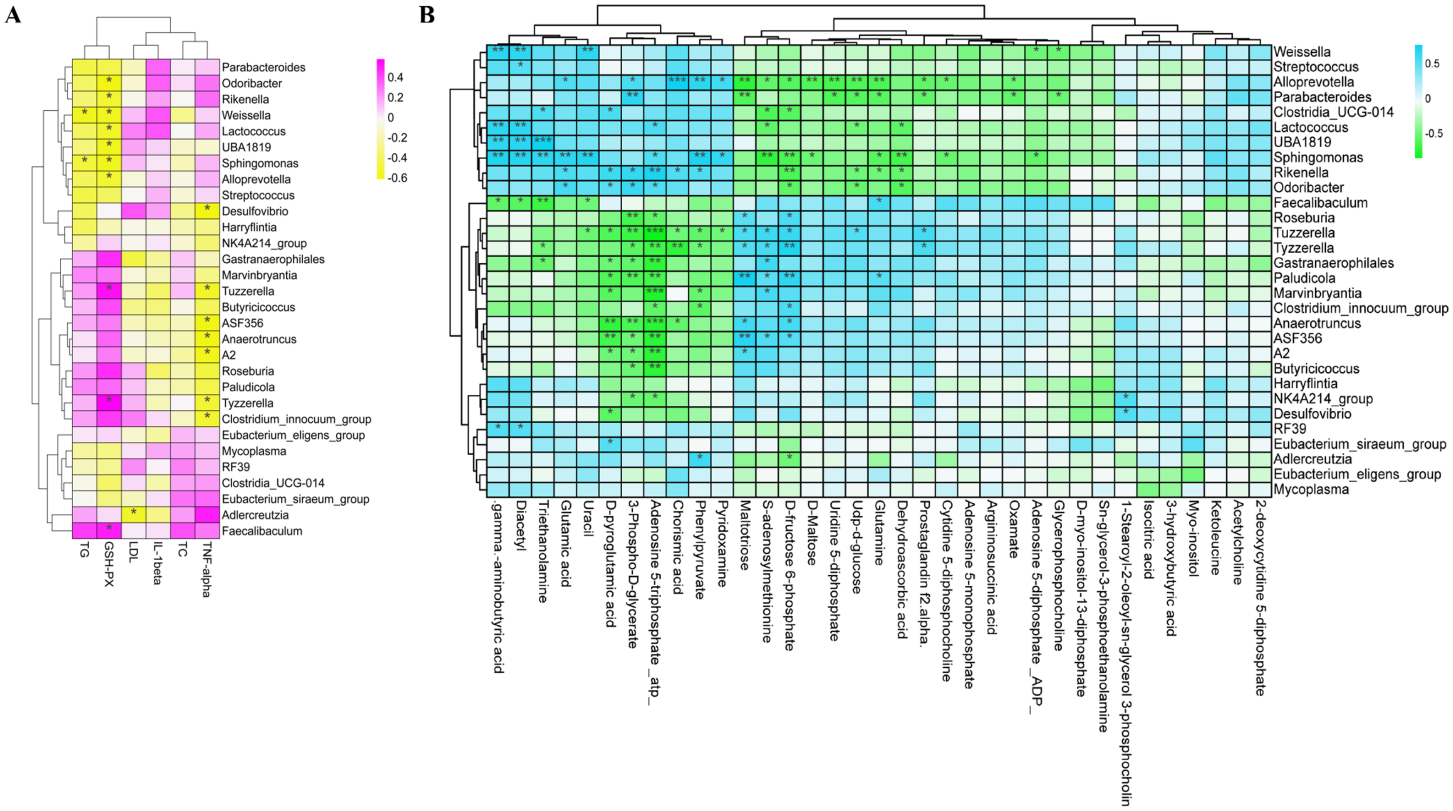
**(A)** Spearman correlation between significantly changed bacterial genera and serum biochemical indices. **(B)** Spearman correlation between significantly changed bacterial genera and liver metabolites (*n =* 6). TG, Triglyceride; GSH-PX, glutathione peroxidase; IL-1β, interleukin-1β; LDL-C, low-density lipoprotein cholesterol; TC, total cholesterol; TNF-α, tumor-necrosis factor-α. *p < 0.05, **p < 0.01, and ***p < 0.001.

To further assess the relationship between gut microbiota and specific metabolites, Spearman’s correlation between significantly affected bacterial genera, metabolites, and serum biochemical indices was analyzed. Serum LDL-C levels were negatively correlated with *Adlercreutzia*, and serum GSH-PX levels were positively correlated with *Faecalibaculum* and *Tyzzerella* genera. Conversely, serum GSH-PX levels were negatively associated with *Lactococcus, Odoribacter*, *Rikenella*, *Sphingomonas*, and *Alloprevotella.* The inflammatory factors IL-1β and TNF-*α* were negatively correlated with *Tyzzerella*, *ASF356*, *Anaerotruncus*, *A2*, and *Clostridium_innocuum_group* (|r| > 0.6, *p* < 0.05). These results indicate that gut microbial diversity was associated with blood biochemical indices and health conditions of the mouse model (Figure 7A).

Furthermore, to estimate the interaction of metabolites with the microbiota, Spearman’s correlation analysis was conducted across 30 significantly different bacterial genera and 35 significantly affected metabolites, which were related to significantly different metabolic pathways. As shown in Figure 7B, a correlation was observed between liver tissue metabolites and gut bacteria. Among these, ATP and 3-phospho-D-glycerate were negatively correlated with increased abundances of *Anaerotruncus, Marvinbryantia*, *ASF356*, *Gastranaerophilales*, *Butyricicoccus*, and *Tyzzerella* but positively correlated with decreased abundances of *Rikenella, Odoribacter*, *and Parabacteroides*. Moreover, glutamine was negatively related to *Rikenella* and *Sphingomonas* but positively correlated with *Faecalibaculum*.

## Discussion

Mulberry fruit contains numerous nutrients and bioactive compounds and exhibits various pharmacological properties, indicating its potential as a functional food for the prevention and treatment of chronic diseases. MA is one of the most abundant plant anthocyanins, and its principal constituent is C3G. Animal, preclinical, and clinical studies have shown that anthocyanins are promising agents for preventing AS. Anthocyanin components in mulberry extracts can scavenge radicals, inhibit LDL oxidation, and decrease atherogenic stimuli in macrophages (28). Oxidized LDL can promote AS through its cytotoxicity, inhibitory effects on macrophage motility, and uptake by macrophage scavenger receptors, thereby increasing cholesterol accumulation and foam-cell formation (29). Preventing the premature death of macrophages improves lipid clearance and inflammatory activity, inhibits LDL oxidation, and reduces macrophage cell death and foam-cell formation. Chang et al. (26) showed the hypolipidemic effects of MA extracts on oleic acid-induced HepG2 cells, and Wu et al. (23) reported that a diet supplemented with MA extracts could protect against body weight gain in HFD-induced mice. In this study, 8 weeks of feeding an HFD induced weight gain and dyslipidemia in ApoE−/− mice. However, MFAE, C3GT, and C3GP interventions resulted in decreased body weight, reduced serum LDL-C and IL-1β levels, and increased GSH-PX levels in mice, supporting the hypolipidemic, anti-inflammatory, and antioxidant effects of MFAE and C3G treatments. Moreover, the results indicated that MA may play an important role in preventing LDL oxidation and lipid accumulation (30), and its intake is negatively correlated with both inflammatory activity and oxidative stress (25).

However, the low bioavailability of anthocyanins in plasma and urine has been reported (17). Limited quantities of anthocyanins are absorbed as hydrolysis products, and numerous ingested compounds enter the colon and interact with the gastrointestinal microbiota. The gut microbiota is crucial for the conversion of dietary anthocyanins into absorbable bioactive substances. MA extracts could reverse intestinal inflammation and oxidative stress and restore the integrity of the intestinal barrier in colitis by changing the structure of gut microbiota. MA intervention reduced *Escherichia/Shigella* abundances but increased *Akkermansia*, *Muribaculaceae*, and *Allobaculum* in colitis mice (31). Moreover, the HFD significantly decreased the abundance of *Lactobacillus* while increasing the abundance of *Oscillibacter*, thereby causing a significant increase in the permeability of the proximal colon (32, 33). In this study, MFAE, C3GT, and C3GP interventions increased the relative abundance of Firmicutes, which may be related to the significantly increased abundances of *Butyricicoccus*, *Anaerotruncus*, and *Tyzzerella* belonging to Firmicutes. *Butyricicoccus*, an autonomous microbe that predominantly colonizes the mucosa-associated surface of the colon (34), exhibits butyrate-generating properties that can reduce intestinal inflammation and prevent cytokine-induced increases in epithelial permeability *in vitro* (35). *Anaerotruncus* species, isolated from two boys with late-onset autism and a fecal sample from an older woman with bacteremia (36, 37), were associated with improved outcomes following fecal transplantation to treat inflammatory bowel disease (38). The abundance of *Anaerotruncus* was also decreased in dextran sulfate sodium-induced UC mice (39), and it was found that probiotic supplementation promoted the blooming of *Anaerotruncus* in antibiotic-treated mice (40). Whether *Tyzzerella* is a pathogen or a probiotic is currently being debated; however, in different models, *Tyzzerella* can play various roles. Fan et al. (41) showed that the Pingwei San treatment for spleen deficiency diarrhea could significantly increase the abundance of *Tyzzerella* in rat feces following treatment. However, another study reported that dietary contamination caused by polystyrene microplastics resulted in an increased abundance of pathogenic *Tyzzerella* (42). Chen et al. (43) reported that a decrease in *Tyzzerella* abundance may be related to the colonization and development of ectopic endometrial lesions and that a low abundance of *Tyzzerella* results in decreased glutamine content in endometriotic lesions. In this study, the abundances of *Anaerotruncus* and *Tyzzerella* were negatively correlated with TNF-*α* but positively correlated with GSH-PX. These results indicated that increased bacterial abundance was associated with increased antioxidant enzyme levels, thereby reducing inflammatory factor levels.

Conversely, MFAE, C3GT, and C3GP interventions decreased the abundances of *Odoribacter*, *Rikenella*, and *Sphingomonas*. In patients with AS and in mice transplanted with proinflammatory microbiota, the abundance of *Odoribacter* species also decreases (44, 45). Firrman et al. (46) reported that supplementation with tomato seed oil reduced the abundance of *Rikenella*. As a gram-negative family, Rikenellaceae is positively associated with LPS production, and its abundance is significantly increased in HFD-fed and aged mice (47–49). Supplementation with anthocyanin-rich blueberry and cranberry extracts reduced plasma LPS concentrations, which were positively correlated with decreased abundances of *Rikenella* and Rikenellaceae bacteria (50), and these findings were consistent with the decreased abundance of LPS biosynthesis proteins and LPS biosynthesis pathways in the C3GP group. The genus *Sphingomonas*, a member of the Sphingomonadaceae family and Alphaproteobacteria class, is associated with sepsis, septic pulmonary embolism, and septic arthritis (51). *Odoribacter*, *Rikenella*, and *Sphingomonas* were negatively correlated with GSH-PX, and a decrease in their abundance could increase antioxidant enzyme levels. These results indicate that changes in the abundance of these bacterial genera may be related to the effects of MA intervention in ApoE−/− mice. MA modulates the gut microbiota, which alters the microbial metabolic functional potential. Through enhanced SCFA synthesis, reduced pro-inflammatory metabolite production, and modulation of metabolic pathways, these microbial changes may induce host responses that may ultimately attenuate AS lesion formation in HFHC-fed ApoE−/− mice.

The microbiota communicates with distal host organs through complex pathways via intestinal microbiota-generated metabolites. It contributes to the metabolic functions of the host (38) and produces biologically active metabolites that affect host receptor stimulation, transmission, and immunomodulatory functions (39). The liver undergoes complex biochemical reactions that occur in response to the metabolism of multiple nutrients, including the oxidation of triglycerides and the synthesis of lipoproteins, cholesterol, and phospholipids. In this study, the effects of MFAE and C3G on liver metabolic parameters were assessed. The increased abundances of *Butyricicoccus*, *Anaerotruncus*, and *Tyzzerella* were negatively correlated with ATP, D-pyroglutamic acid, and 3-phospho-D-glycerate, but positively correlated with D-fructose-6-phosphate, maltotriose, and S-adenosylmethionine. Extracellular ATP regulates various biological processes, including cardiac function, vasodilation, ion channel function, DNA synthesis, cell division, neurotransmission, muscle contraction, bone metabolism, liver glycogen metabolism, and inflammation (52). Extracellular ATP can exert its proinflammatory activity in various ways through plasma membrane purinergic receptors. These include the enhanced production of inflammatory mediators, monocyte chemoattractant protein-1, and growth-regulated oncogenes and the activation of cell adhesion molecules in endothelial cells (53). In this study, ATP peak value reductions, which are essential for limiting an inflammatory stimulus, occurred with the MFAE, C3GT, and C3GP treatments. MFAE treatment also decreased the level of ADP, another potent inflammatory metabolite that regulates platelet reactivity at vascular injury sites and reduces its subsequent hydrolysis to AMP and inhibition of platelet aggregation via adenosine (54). Therefore, the reduction in ADP concentration with MFAE treatment was an additional anti-inflammatory process. These results were consistent with the significantly reduced IL-1β levels by C3GT and TNF-*α* levels by MFAE, as well as with the increased liver metabolites, adenosine, and inosine.

Furthermore, decreased bacterial genera (*Odoribacter*, *Rikenella*, and *Sphingomonas*) were positively correlated with glutamic acid levels, which are related to 40 metabolic pathways, but negatively associated with glutamine levels. MFAE, C3GT, and C3GP treatments increased the levels of the liver metabolite glutamine; however, glutamic acid and D-glutamic acid levels decreased following treatment. Glutamine is the most abundant and versatile amino acid in the body and plays a critical role in nitrogen exchange among organs, intermediary metabolism, immunity, and pH homeostasis (55, 56). Glutamine may prevent aortic AS in a hyperlipidemia-induced mouse model, and its protective mechanism may be associated with the downregulation of glycolysis, O-GlcNAc, oxidative stress, and proinflammatory pathways (57). It can be converted to glutamate and α-ketoglutarate via glutaminase and glutamate dehydrogenase, respectively. Glutamine also supports the normal immunological structure and function of the gastrointestinal tract. In animal studies, glutamine deprivation has been associated with decreased bacterial genera by affecting glutamine-related metabolic pathways (e.g., purine metabolism), thereby reducing oxidative stress and arterial inflammation.

In contrast, the pathway enrichment analysis revealed that ATP was related to 21 different pathways, including the lysosome pathway, purine metabolism, alanine, aspartate, and glutamate metabolism, neuroactive ligand–receptor interaction, the synaptic vesicle cycle, and metabolic pathways. The lysosomal pathway was shared and downregulated by MFAE, C3GT, and C3GP treatments. Lysosomes break down several biomolecules and organelles, and the malfunction of the lysosomal compartment plays a significant role in the etiology and pathogenesis of AS. These mechanisms affect AS progression, including inflammation, efferocytosis, exocytosis, autophagy, mTOR signaling, and iron metabolism (58). Notably, mTOR inhibitors exert unique anti-AS effects, including the induction of autophagy, depletion of plaque macrophages, activation of cholesterol efflux, and inhibition of proinflammatory signaling (59, 60). Moreover, inhibiting mTOR signaling can reduce HIF-1α expression, thereby enhancing the function of glutamine and glucose metabolism pathways (61). Changes in purine metabolism include increases in anti-inflammatory metabolite inosine and adenosine concentrations and decreases in the inflammatory metabolites ATP and ADP. Notably, ATP, ADP, and adenosine are actively involved in cellular signaling cascades, and several polyphenols affect the turnover of these metabolites by regulating energy metabolism-related enzymes (54).

Vitamin B6 and *β*-alanine metabolism were also affected following MA intervention. In particular, MFAE, C3GT, and C3GP treatments significantly changed the metabolic activity of these metabolites, as demonstrated through the gut microbial functional prediction and liver metabolite enrichment analyses. Two significant derivatives of vitamin B6 are the coenzyme species pyridoxamine-5-phosphate and pyridoxal-5-phosphate (PLP). Notably, PLP is involved in the metabolic pathways of neurotransmitters and the metabolism of amino acids, lipids, and carbohydrates (62). PLP also participates in other significant pathways related to immune function (63), thrombosis (64), and inflammation (65), all of which are crucial at every stage of the AS process. Therefore, we hypothesize that the therapeutic benefits of MA in AS are linked with the above metabolic pathways.

Notably, this research was limited to capturing the microbial and liver metabolic changes. To reveal the precise effects of MA on AS progression, we need to quantify the inhibition rate of aortic plaque area and further evaluate potential mechanisms, including atherosclerosis-related genes, enzymes, and protein expression, especially genes related to the above enriched pathways.

## Conclusion

This study provides evidence for dietary mulberry anthocyanin extract, not only the purified single component (C3G) but also the semi-purified anthocyanin mixture (MFAE), decreasing the inflammatory factors related to the gut microbiota of HFHC diet mice, and may potentially serving as a strategy to improve host health, and that following the extension of the intervention period, the number of changed gut microbiota and liver metabolites was increased. MA intervention may attenuate AS-associated risk factors by attenuating the gut microbiota, which is significantly associated with blood biochemical indices and liver metabolites, especially ATP and glutamine. The mechanism by which MA attenuates AS may involve regulation of the lysosome pathway, thereby reducing arterial oxidative stress and inflammatory factors. However, functional validation experiments are essential to confirm the mechanistic roles of these key microbial and metabolic factors.

## Data Availability

The 16SrRNA sequencing data have been deposited at the SRA of the NCBI database under project identification number PRJNA1131616. The Metabolomics data have been deposited in the EMBL-EBI MetaboLights database (DOI: 10.1093/nar/gkad1045, 37971328) with the identifier “MTBLS14060”; other data that support the findings of this study are available in the Supplementary material of this article.

## Glossary

ACON: control
ADP: adenosine-5̓ -diphosphate
AMP: adenosine-5̓ -monophosphate
ANOVA: analysis of variance
AS: atherosclerosis
ASV: amplicon sequence variant
ATP: adenosine-5̓ -triphosphate
C3G: cyanidin-3-glucoside
C3GP: C3G group 2
C3GT: C3G group 1
C3R: cyanidin-3-rutinoside
CAC: coronary artery calcium
CVD: cardiovascular disease
ELISA: enzyme-linked immunosorbent assay
GSH-PX: glutathione peroxidase
HCM: hypertrophic cardiomyopathy
H&E: hematoxylin and eosin
HFD: high-fat diet
HFHC: high-fat and high-cholesterol diet
HPLC–MS: high-performance liquid chromatography-mass spectrometry
IL-1β: interleukin-1β
KW: Kruskal-Wallis
KEGG: Kyoto Encyclopedia of Genes and Genomes
LDA: linear discriminant analysis
LDL: low-density lipoprotein
LDL-C: low-density lipoprotein cholesterol
LC-MS: liquid chromatography-mass spectrometry
MA: mulberry anthocyanin
MFAE: mulberry fruit anthocyanin extract
OPLS-DA: orthogonal partial least squares-discriminant analysis
P3G: pelargonidin-3-glucoside
PCA: principal component analysis
PCR: polymerase chain reaction
PICRUSt: phylogenetic investigation of communities by reconstruction of unobserved states
PLP: pyridoxal-5-phosphate
SD: standard deviation
TC: total cholesterol
TG: triglyceride
TNF-*α*: tumor necrosis factor-α
TMAO: trimethylamine-N-oxide
