# Supplementary material for: Ameliorative effects of mulberry fruit anthocyanin extract on gut microbiota and liver metabolites in high-fat and high-cholesterol diet-fed ApoE−/− mice
Source: Front Nutr. 2026 Mar 18;13:1780996. doi: 10.3389/fnut.2026.1780996 (PMC13040788; doi:10.3389/fnut.2026.1780996)
Supplement: Supplementary file 1 [file supplementary_file_1.docx]

**Supplementary figures**


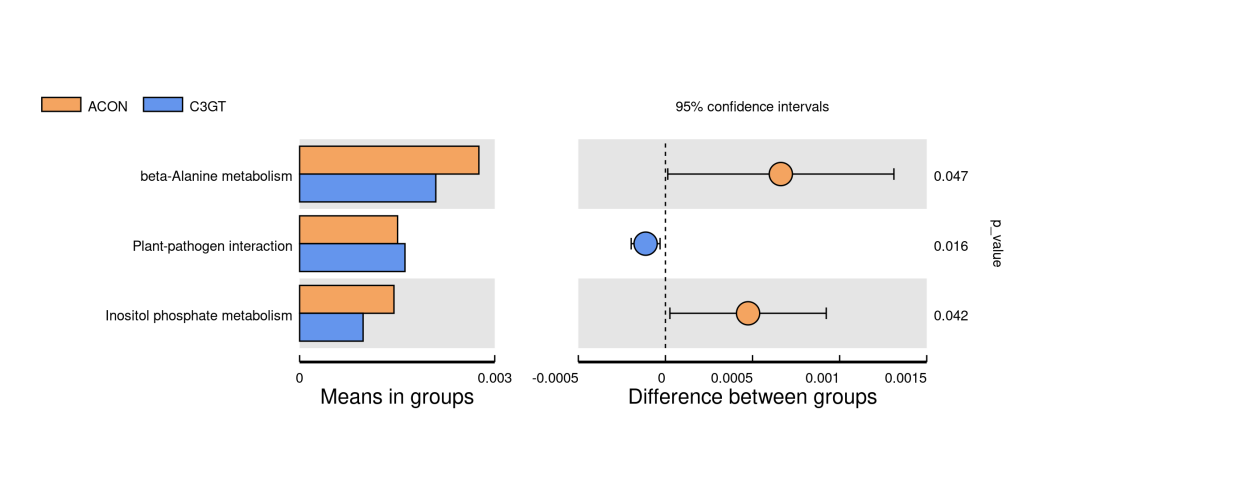

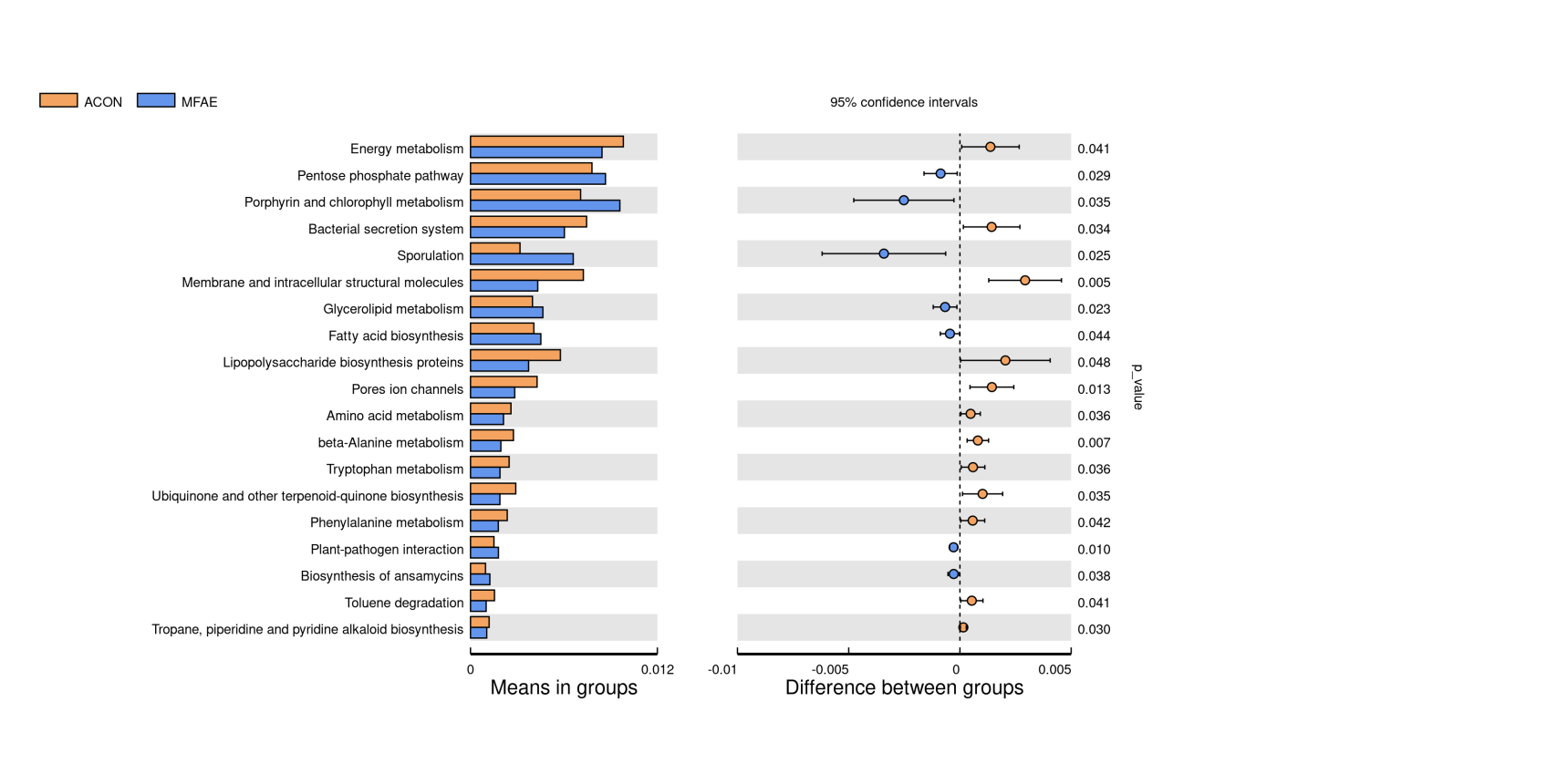


B

A


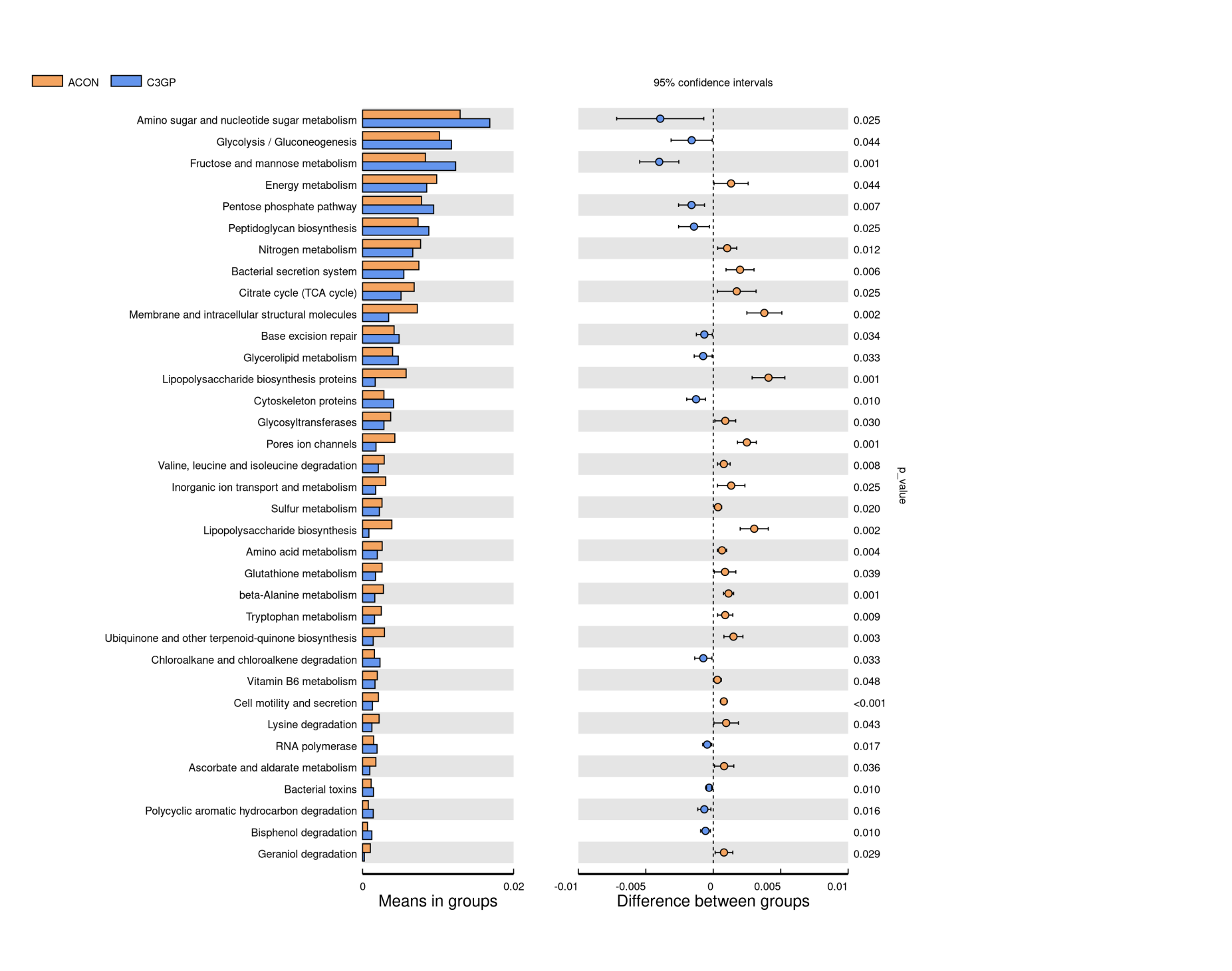


C

**Figure S1.** Significantly different pathways between groups. A, different pathways between ACON and MFAE; B, different KEGG pathways between ACON and C3GT; C, different KEGG pathways between ACON and C3GP.


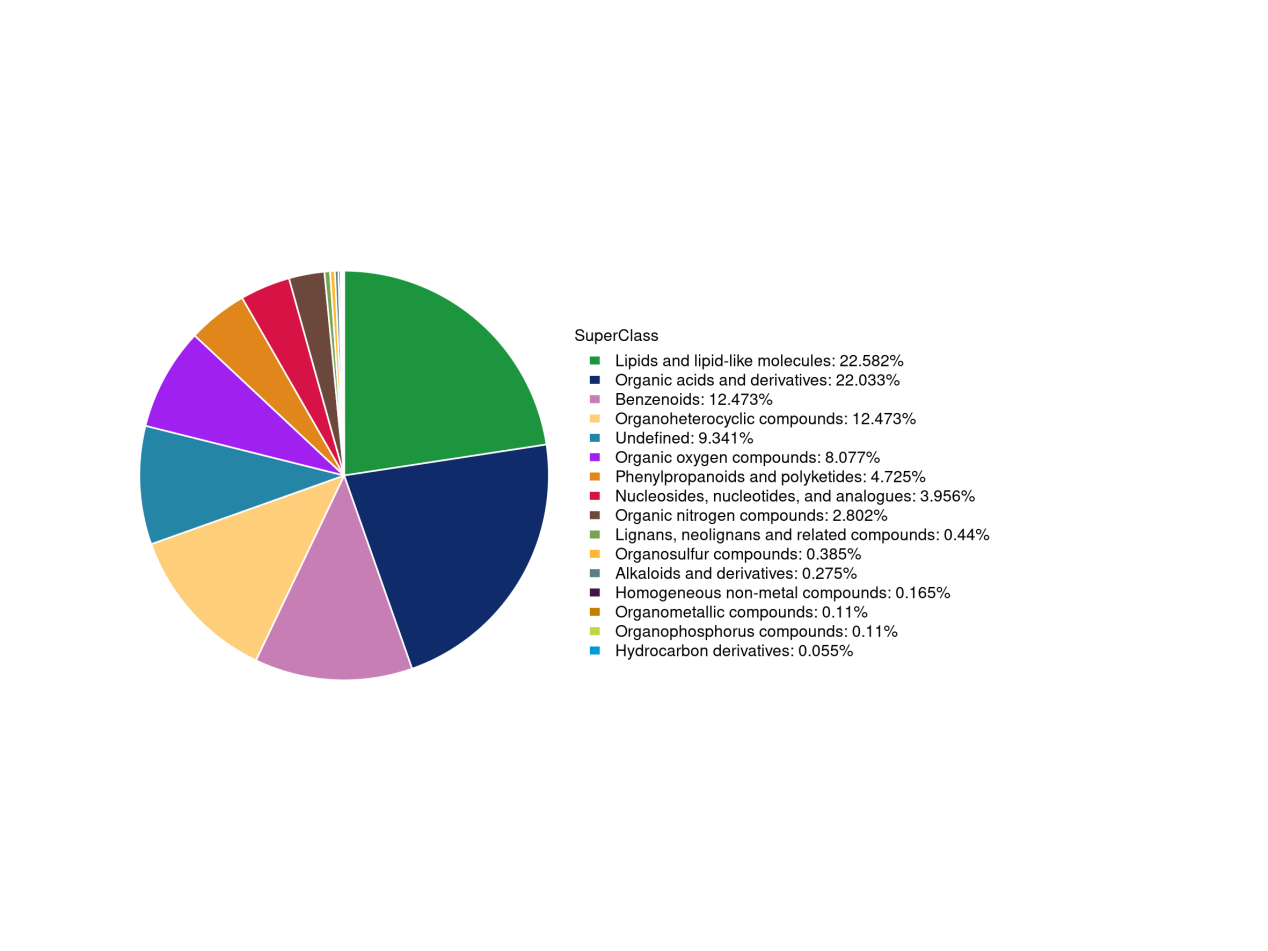

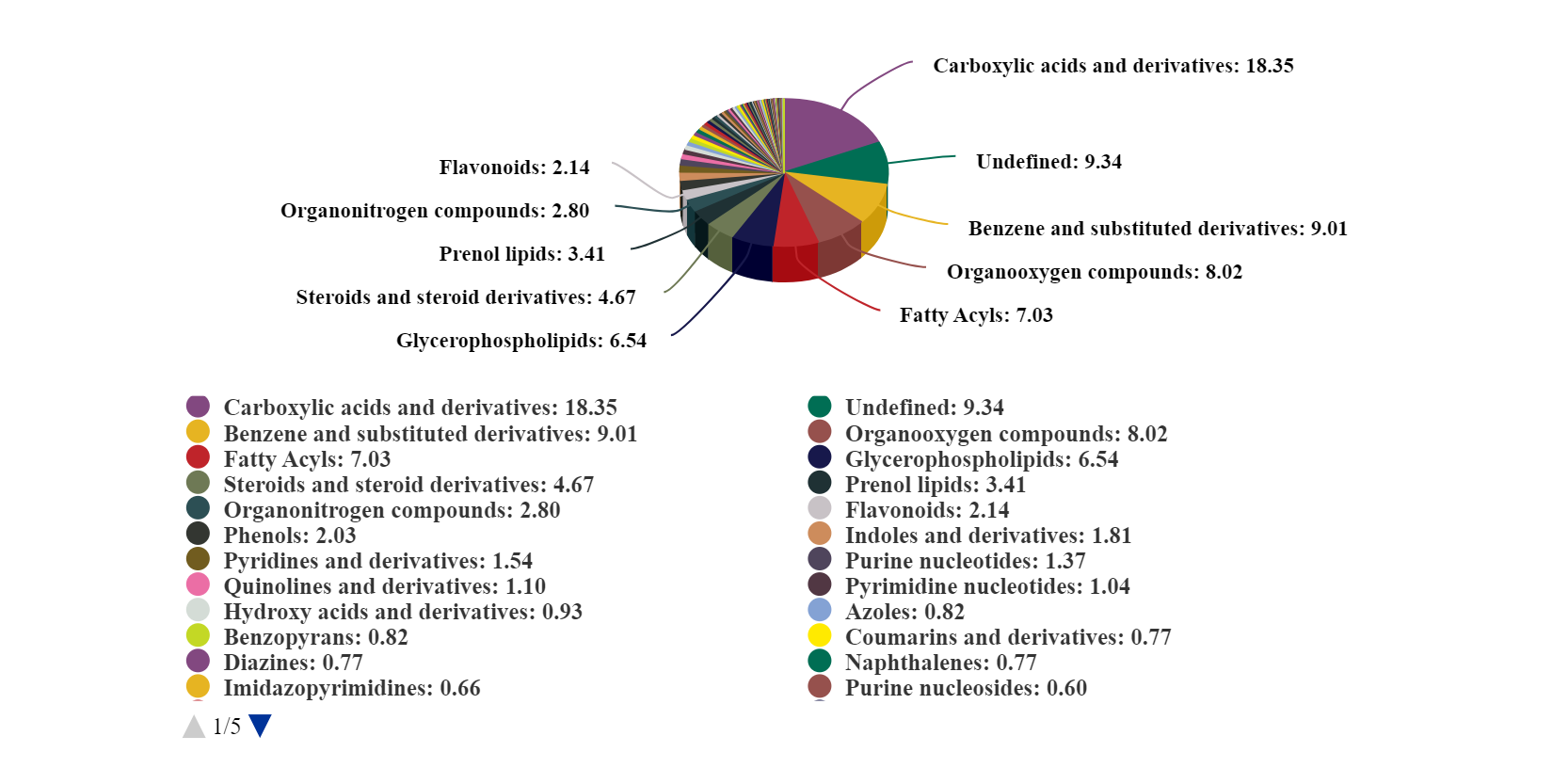


B

A

**Figure S2.** A, Liver metabolites proportion on superclass level; B, Liver metabolites proportion on class level.

**Figure S3.** Principal component analysis (PCA) of different samples in positive ion

ACON MFAE

ACON MFAE

ACON C3GT

ACON C3GT

ACON C3GP

ACON C3GP

F

E

D

C

A

B

Positive

Negative


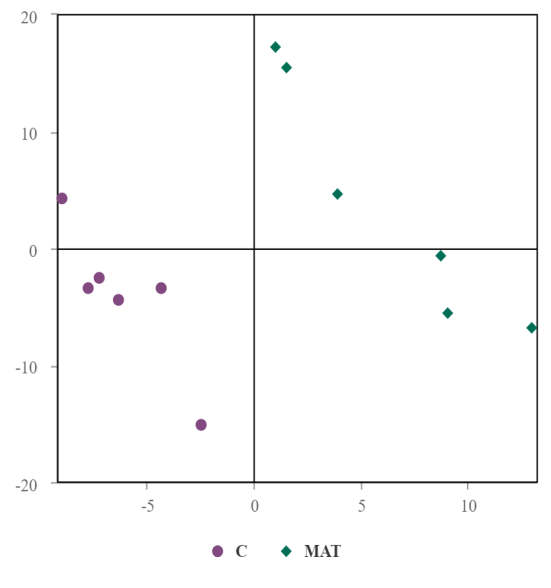

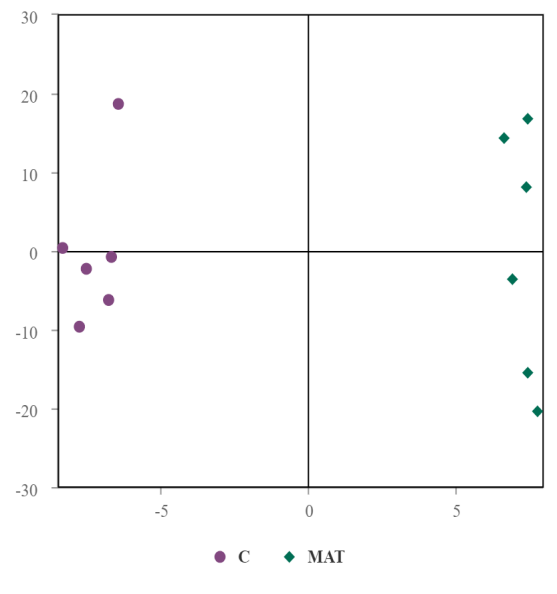

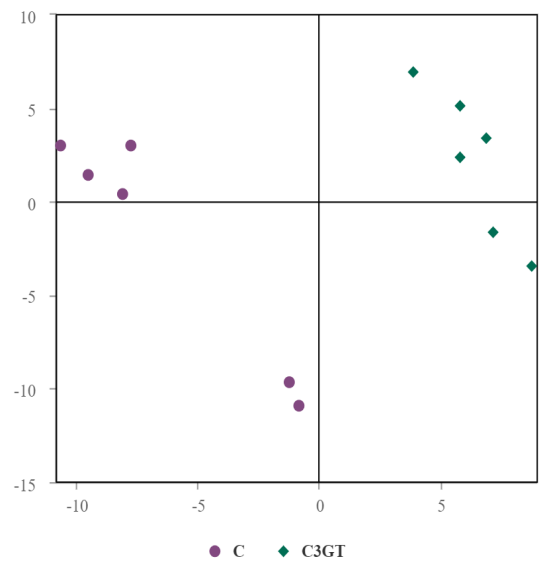

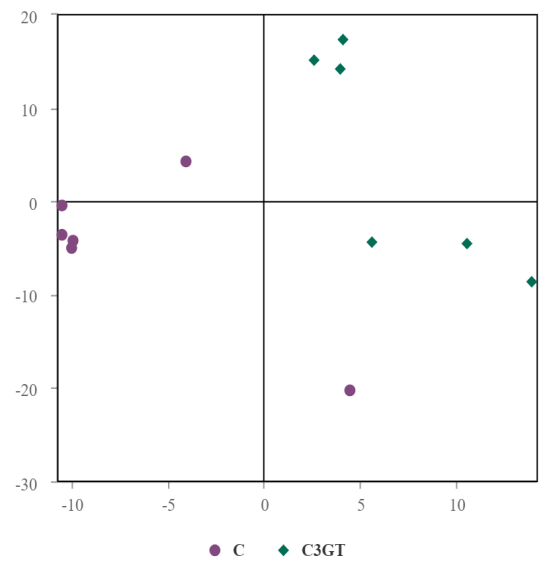

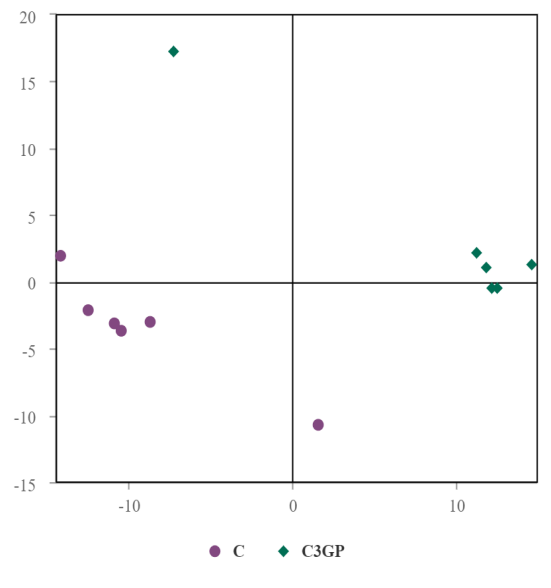

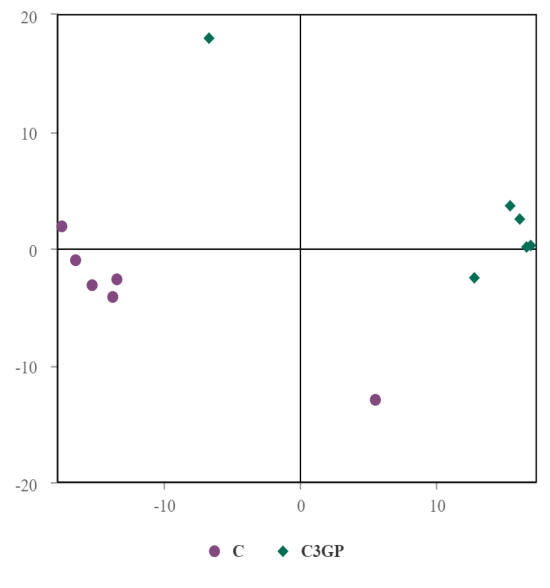


mode (A, C, E) and negative ion mode (B, D, F).


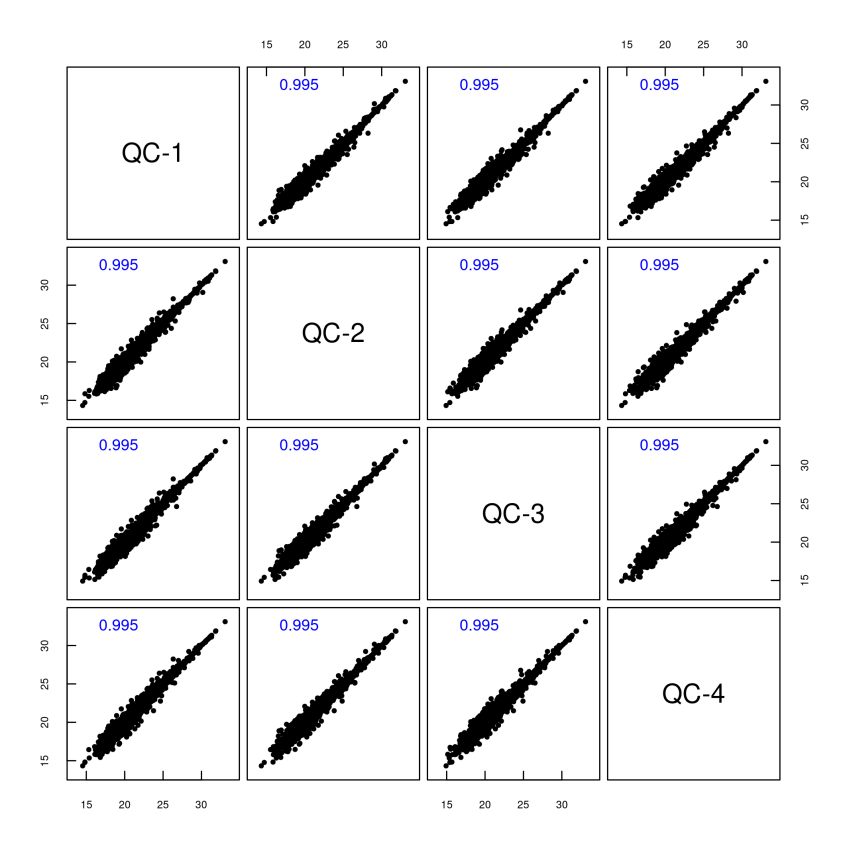

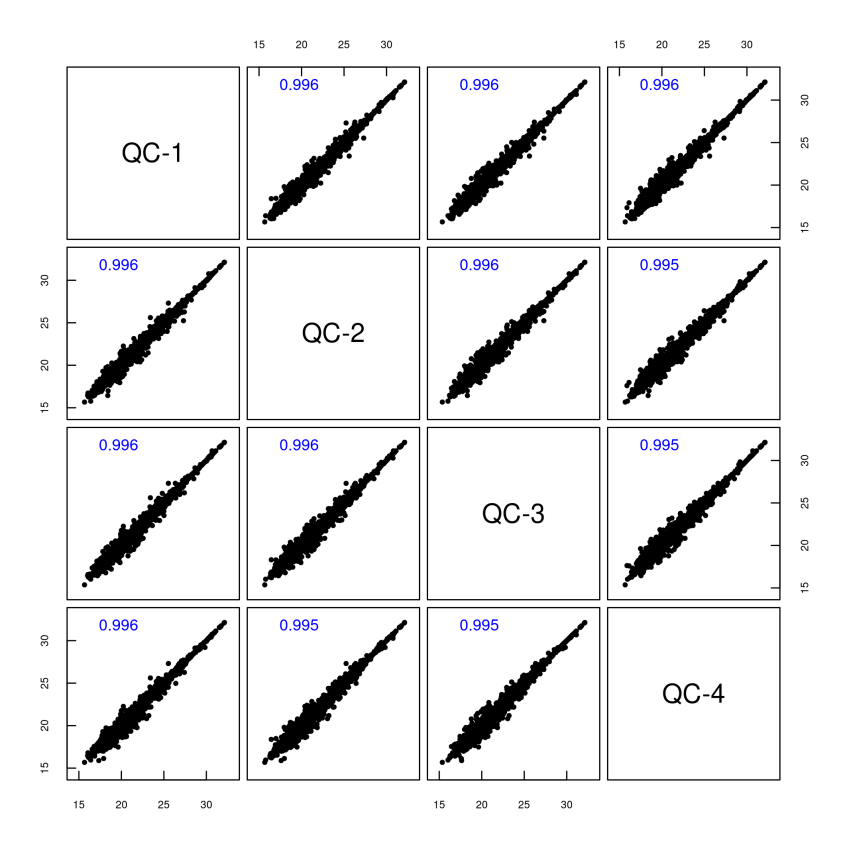


A

B

**Figure S4.** QC sample correlation in positive ion mode (A) and negative ion mode (B).


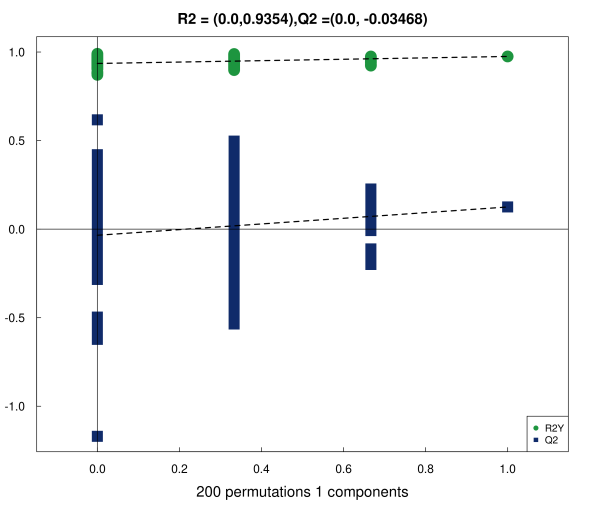

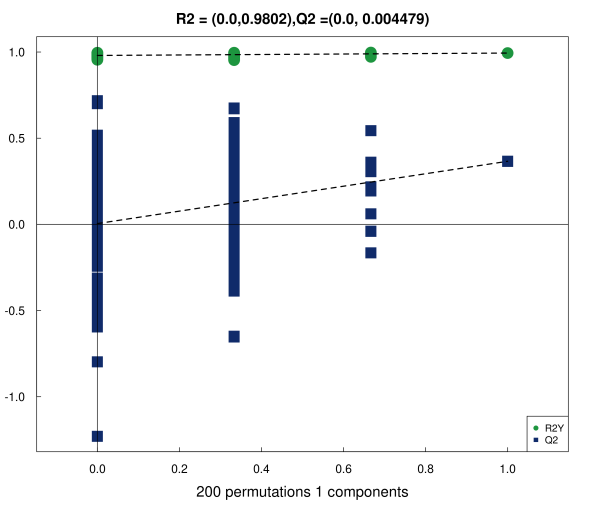


A

B


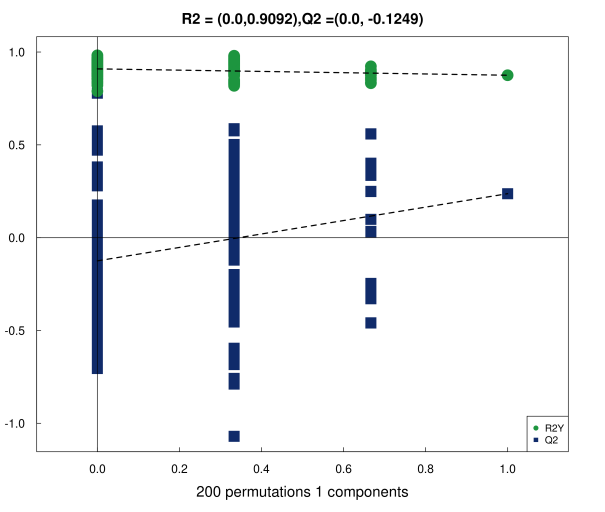

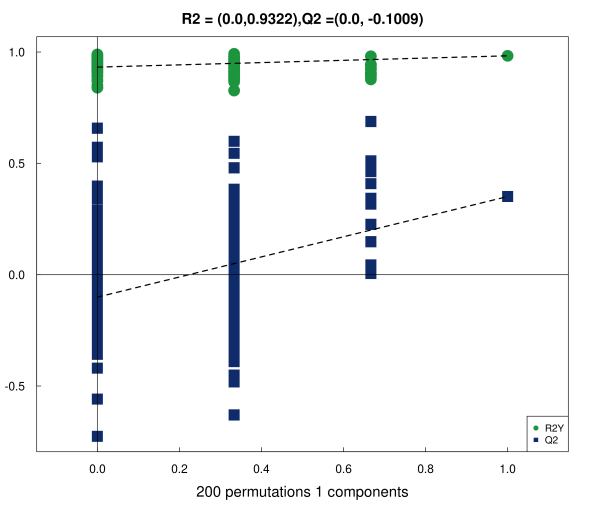

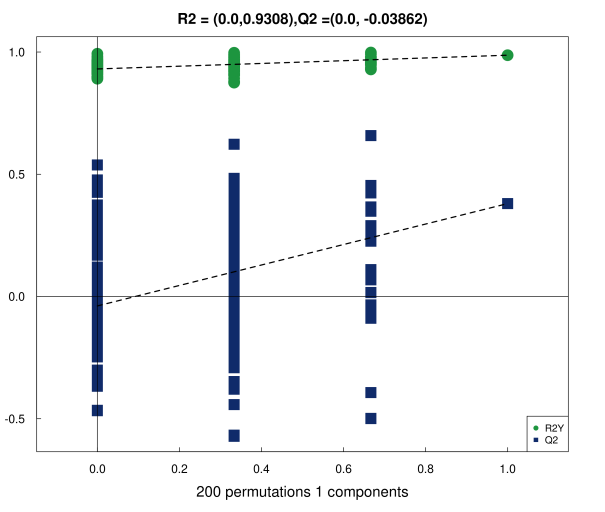

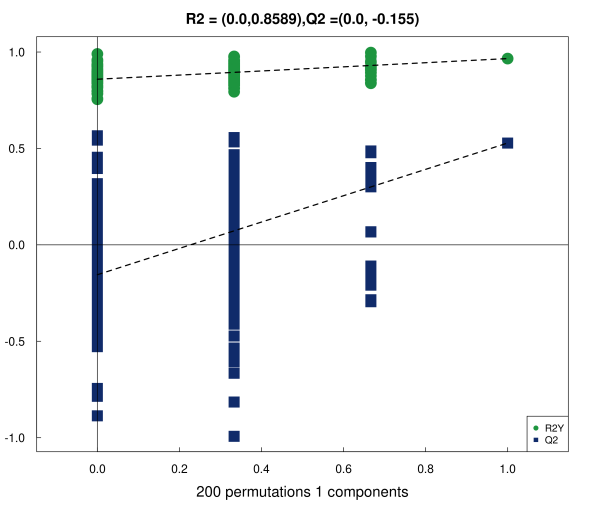


F

E

D

C

**Figure S5.** Permutation plot for ACON and MFAE groups by the 200-response reciprocity test in the positive ion mode (A) and negative ion mode (B); Permutation plot for ACON and C3GT groups by the 200-response reciprocity test in the positive ion mode (C) and negative ion mode (D). (I) Permutation plot for ACON and C3GP groups by the 200-response reciprocity test in the positive ion mode (E) and negative ion mode (F).


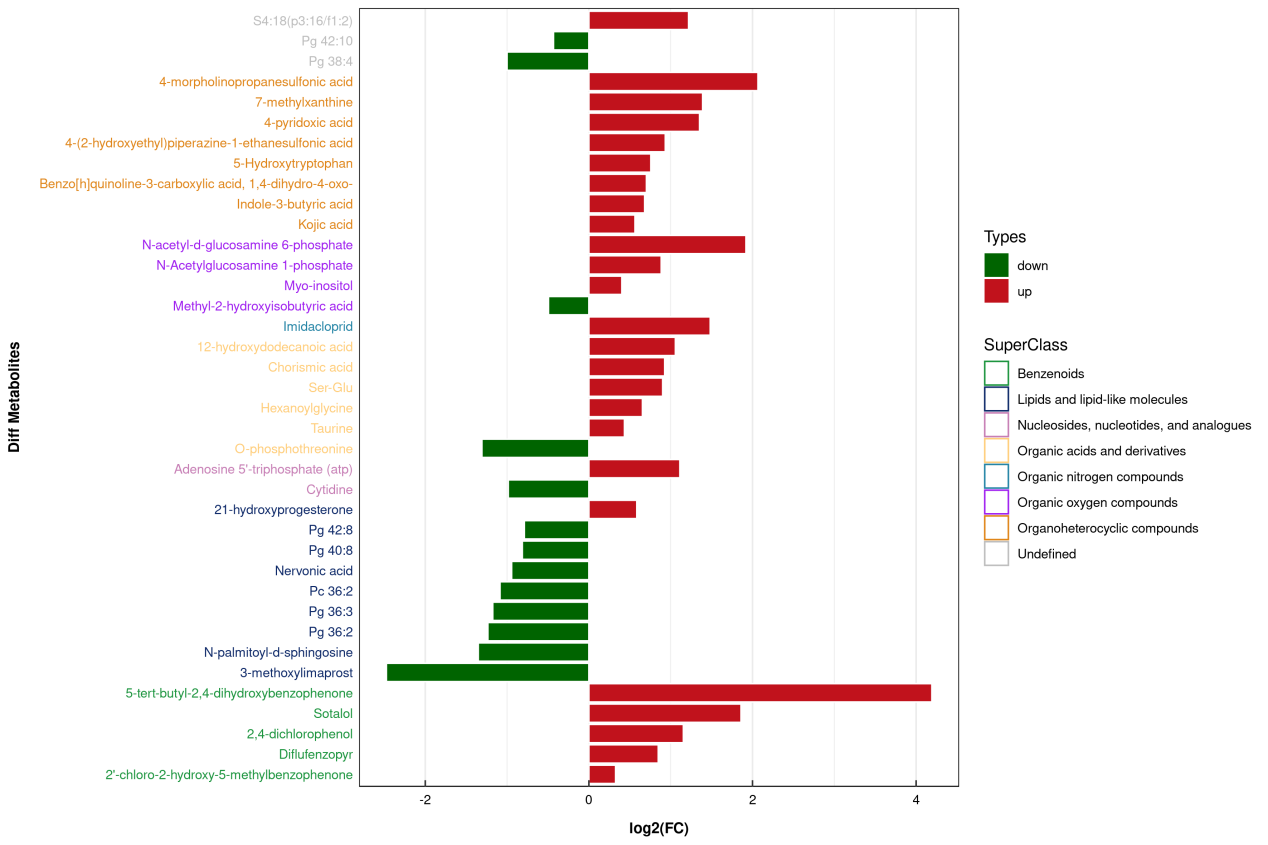

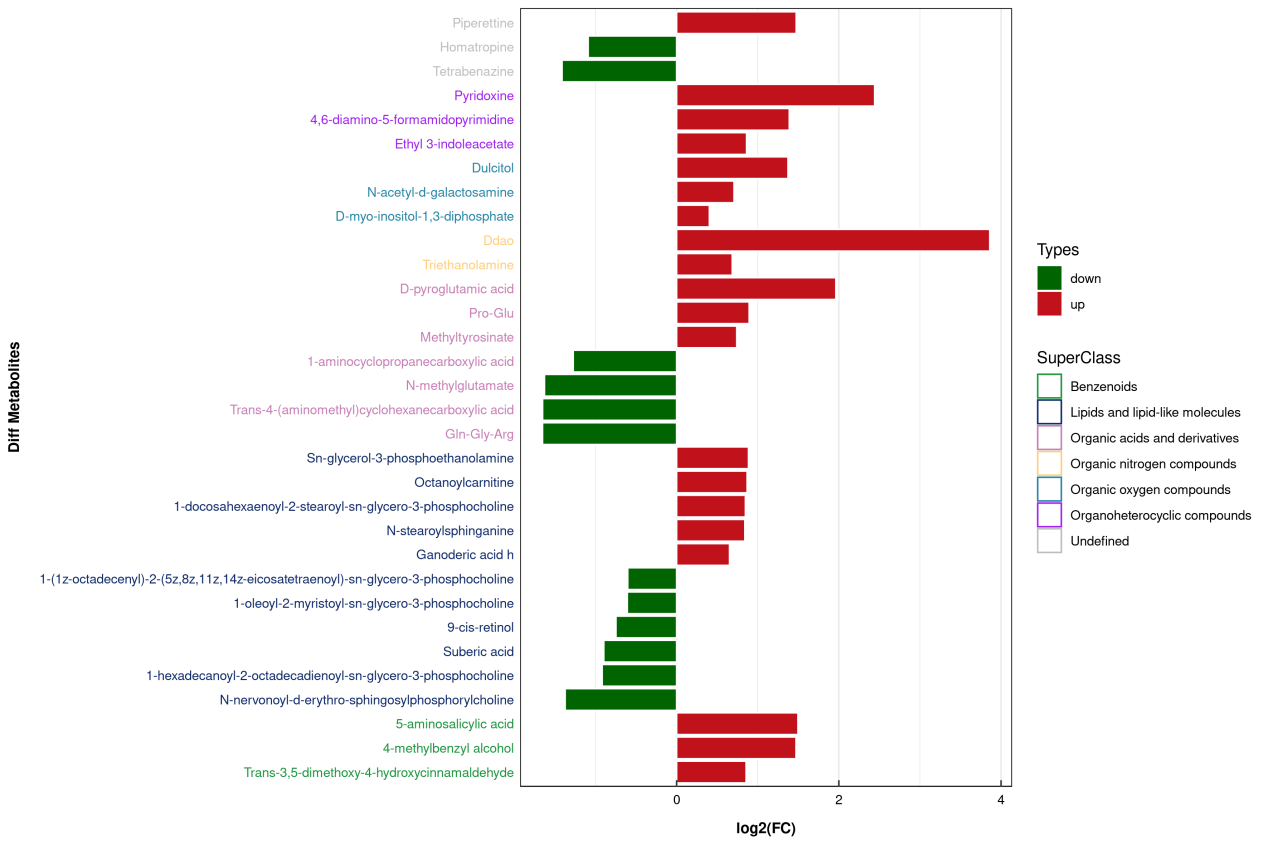


A


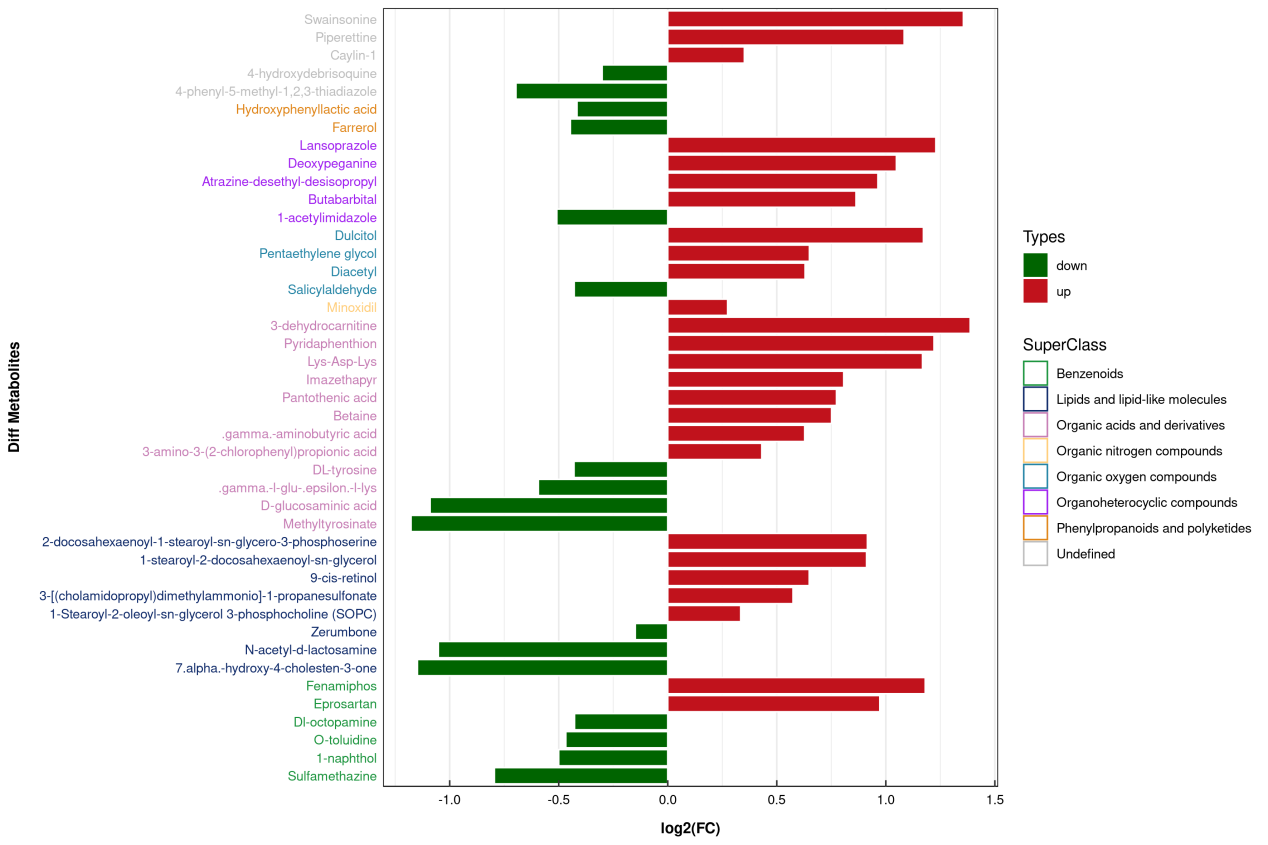

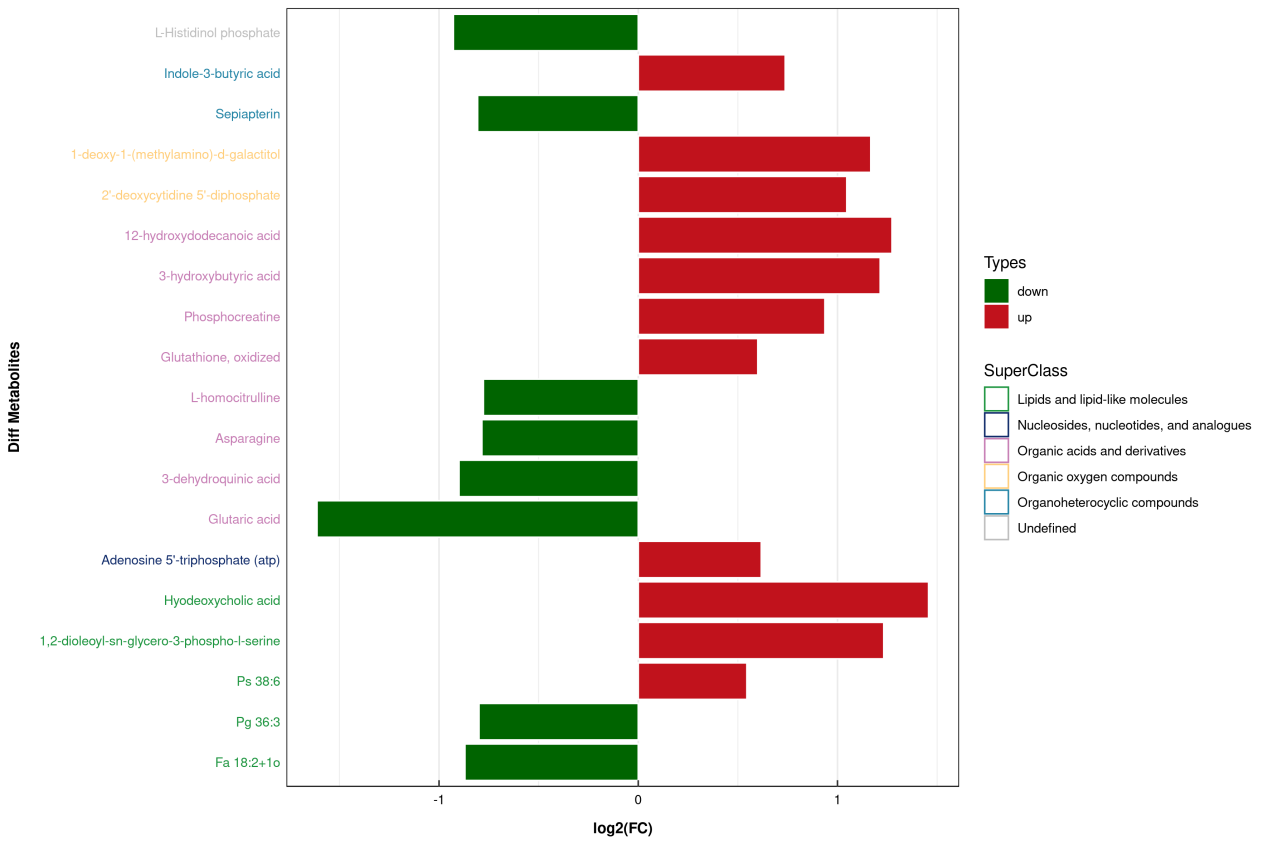


B

D

C


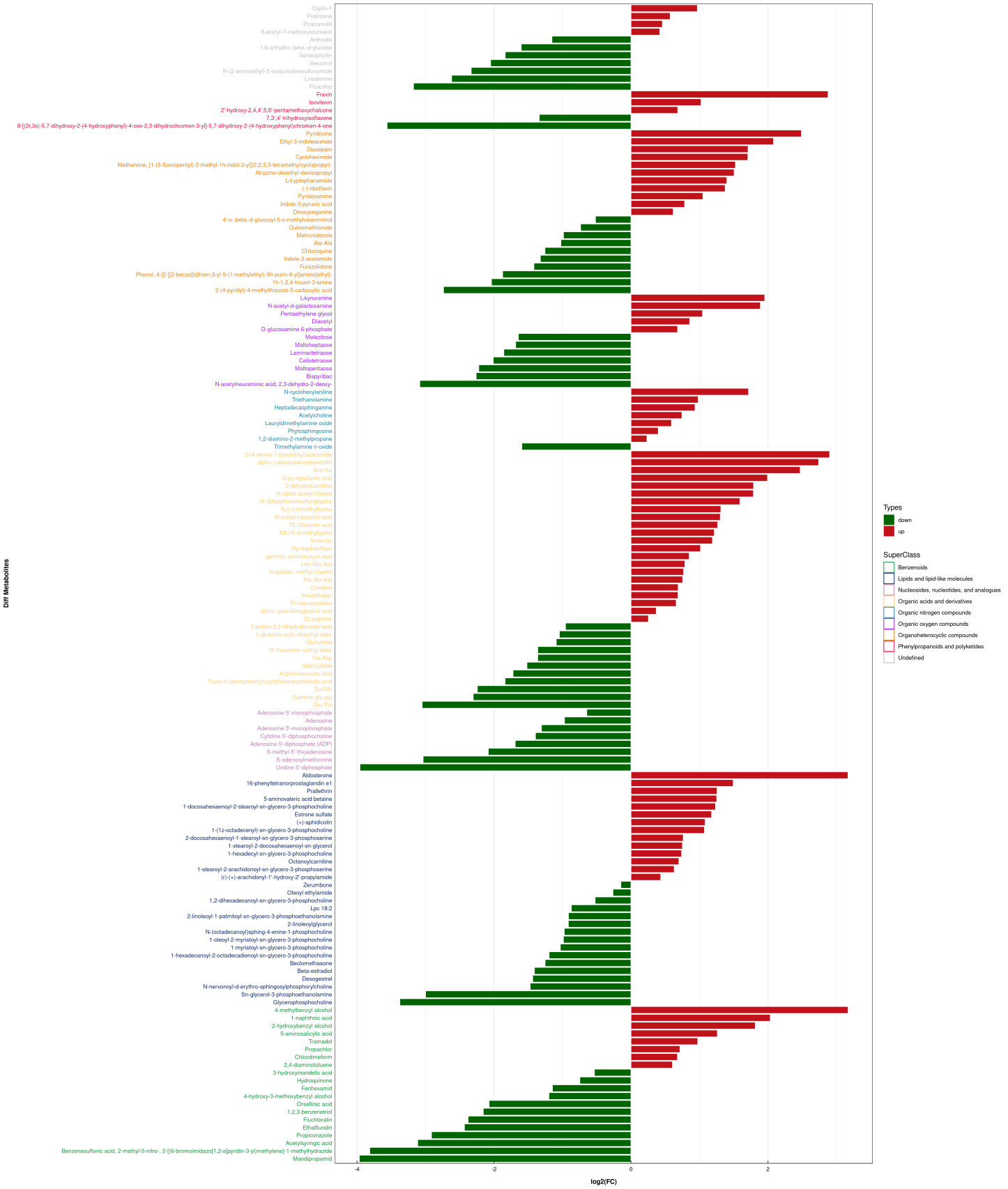

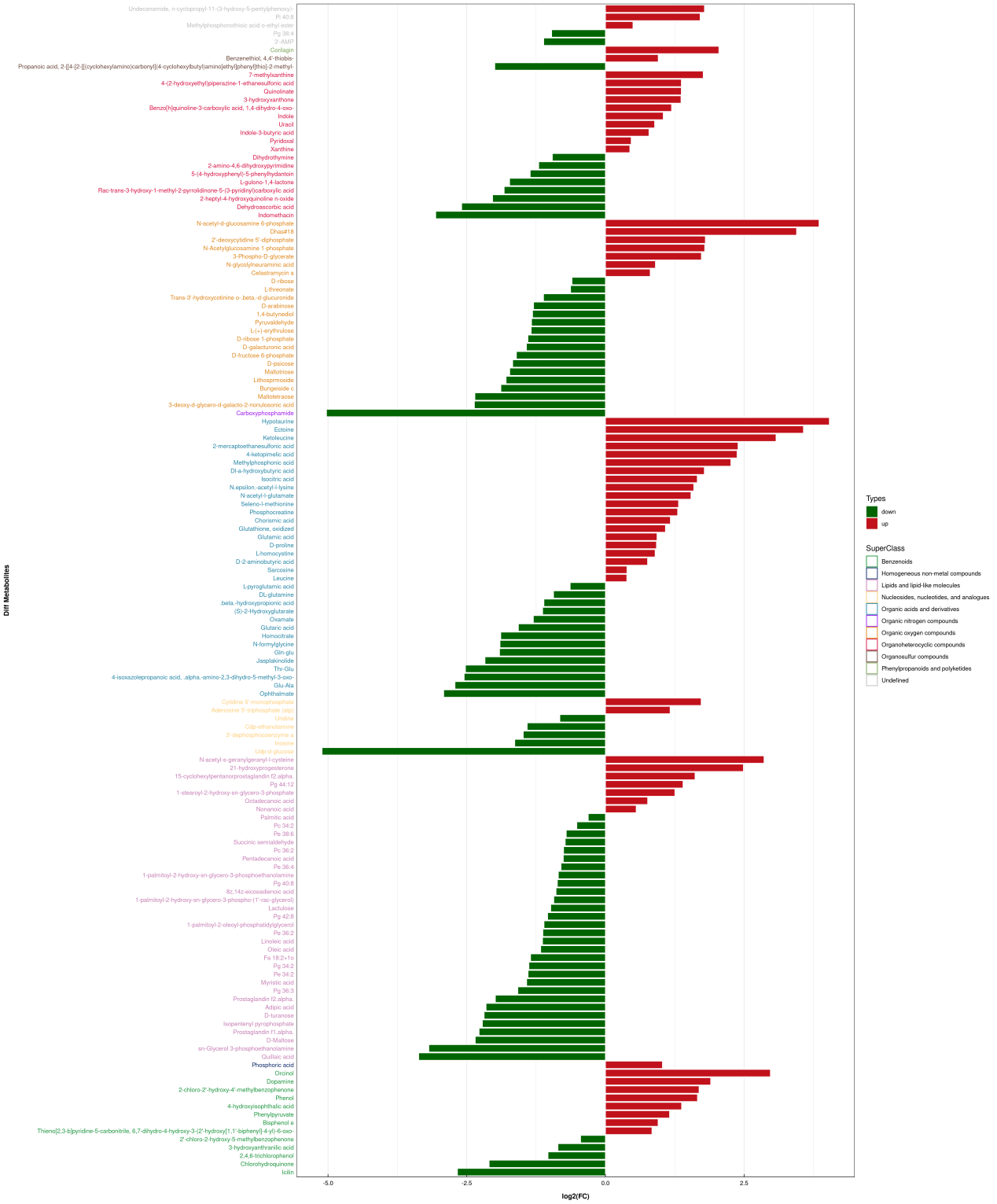


E

F

**Figure S6.** Differential metabolites between groups, ACON compared with MFAE, C3GT, and C3GP groups, respectively. A,C,E were different metabolites on positive ion mode, B,D,F were different metabolites in negative ion mode.


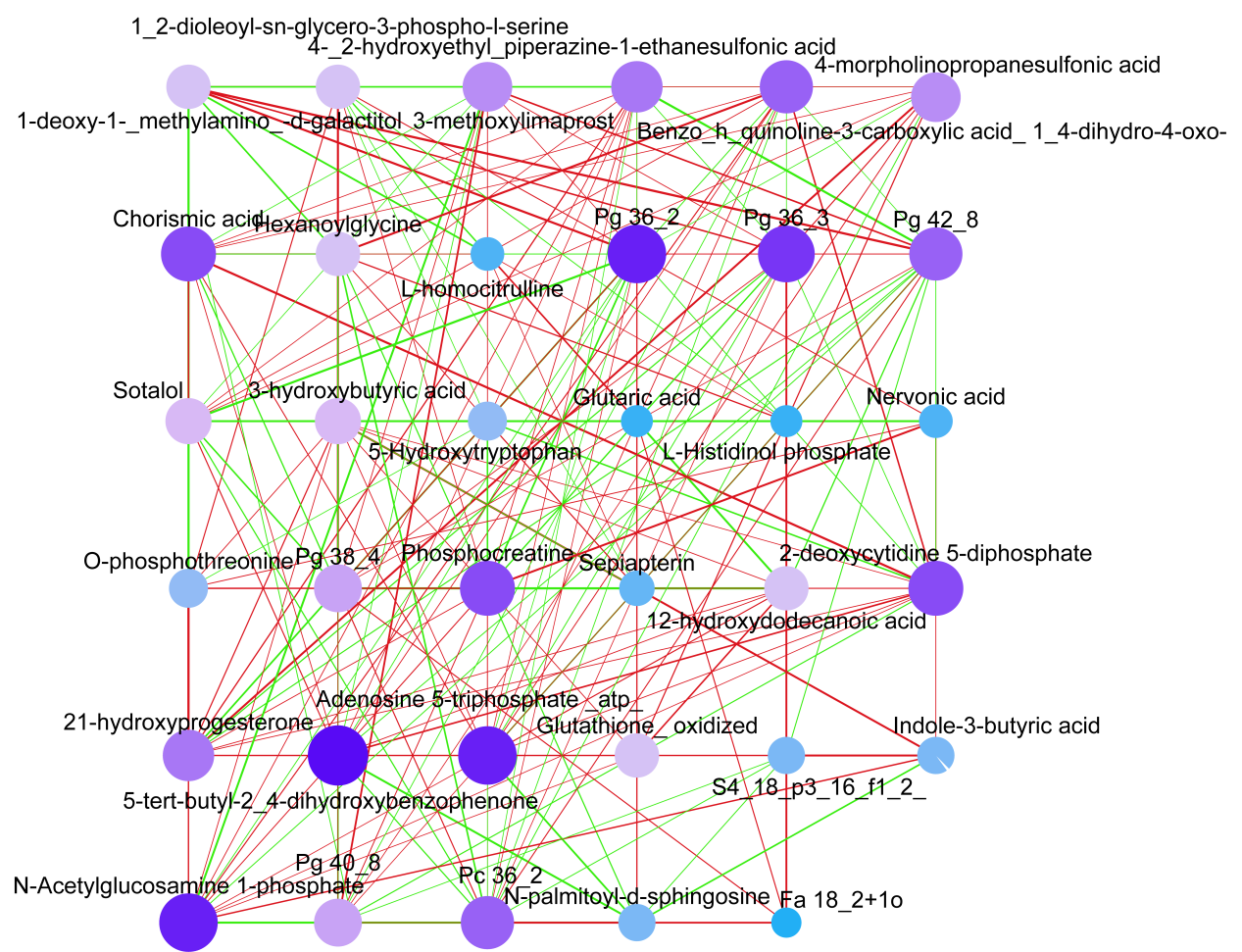


**Figure S7.** Spearman correlation analysis of 35 significantly differential metabolites between groups. Green line, negative correlation, red line positive relation, line width indicates the P value. Circle size and color correspond to metabolite peak area; the bigger the circle, the higher the peak area.


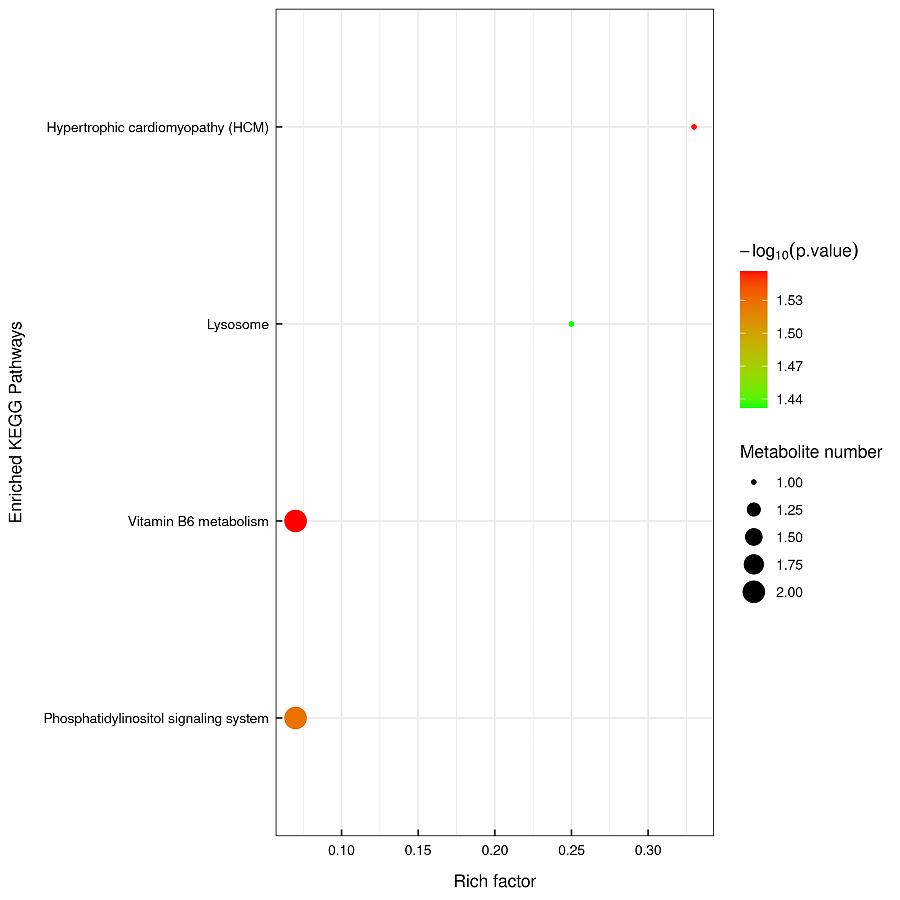

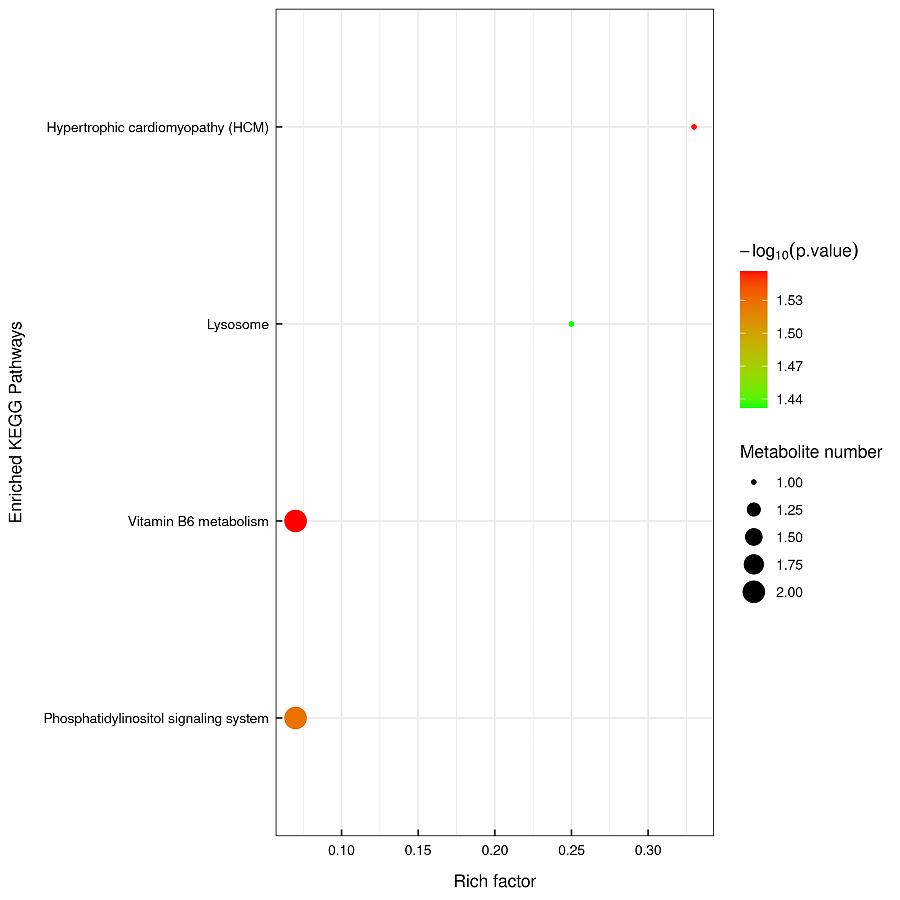

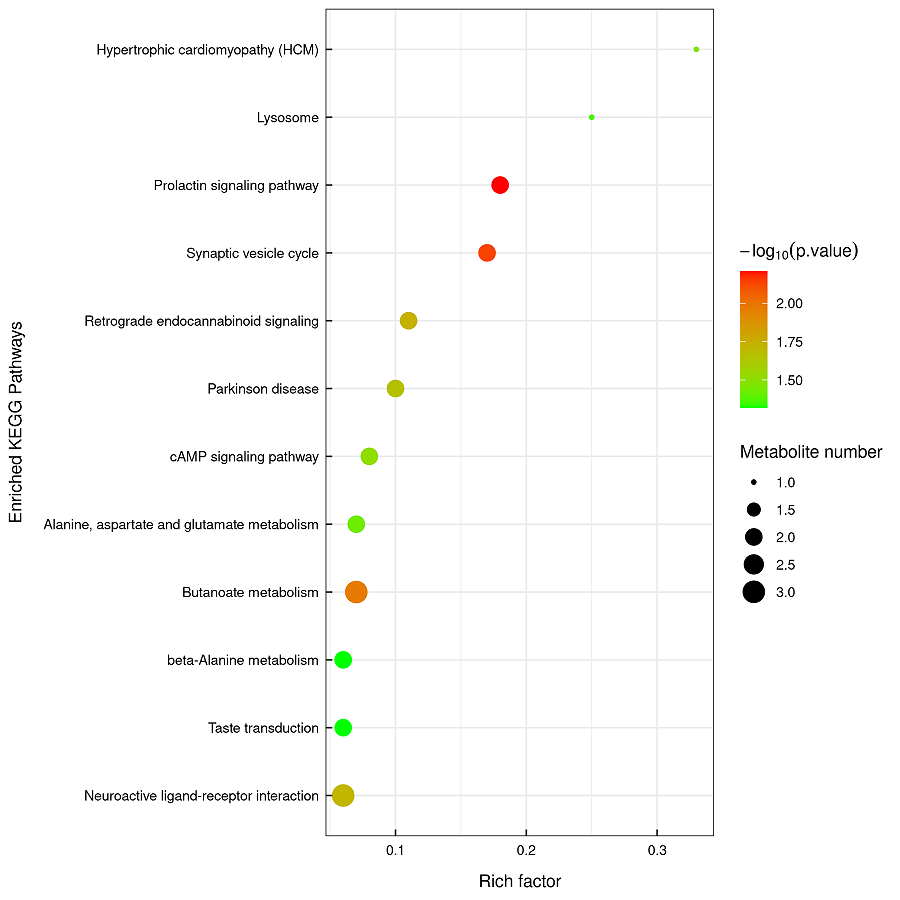


B

A

**Figure S8.** Pathway enrichment analysis of liver metabolites. A, ACON compared with MFAE; B, ACON compared with C3GT.

Supplementary tables

Table S1 The content and fragment information of MFAE.

| Chemical compound | Abbreviation | Molecular ion (m / z) | Fragment ion (m / z) |
| --- | --- | --- | --- |
| Cyanidin3-O- (6 ″ -O rhamnose-galactoside) | C3RGa | 595 | 449/287 |
| Cyanidin-3-O- (6 ″ -O rhamnose-glucoside) | C3R |  | 449/287 |
| Cyanidin-3-(2G-glucorutinoside) | C3-2G-gluco | 757 | 287 |
| Cyanidin-3,5-diglucoside | Cy-3, 5-diglu | 611 | 449/287 |
| Cyanidin-3-sophoroside | Cy-3-sop |  | 287 |
| Delphinidin-3-(coumaroyl)glucoside | Dp-3-Co |  | 465/303 |
| Delphinidin-3-rutinoside | Dp3R |  | 465/303 |
| Cyanidin-7-glucoside | C7G | 449 | 287 |
| Cyanidin-3-galactoside | C3Ga |  | 287 |
| Cyanidin-3-glucoside | C3G |  | 287 |
| Delphinidin-3-rutinoside 5-glucoside | D3R5-gluco | 773 | 611/303 |
| Delphinidin-3-(malonyl)glucoside | D3M-gluco | 551 | 303 |
| Paeoniflorin-3-xyloserhamnoside | P3X | 579 | 301 |
| Pelargonidin-3-rutinoside | P3R |  | 433/271 |
| Pelargonidin-3-(coumaroyl)glucoside | Pl-3-Co |  | 433/271 |
| Pelargonidin-3-glucoside | P3G | 433 | 271 |
| Morning glory-3-glucoside | Pt3G | 479 | 317 |
